# Range of invasive meningococcal disease sequelae and health economic application – a systematic and clinical review

**DOI:** 10.1186/s12889-022-13342-2

**Published:** 2022-05-31

**Authors:** Jing Shen, Najida Begum, Yara Ruiz-Garcia, Federico Martinon-Torres, Rafik Bekkat-Berkani, Kinga Meszaros

**Affiliations:** 1 GSK, Avenue Fleming, 20 1300 Wavre, Belgium; 2 Present address: Takeda Pharmaceutical Company Limited, Zurich, Switzerland; 3 GSK, Freelance C/O GSK, Wavre, Belgium; 4 GSK, Rockville, USA; 5 Genetics, Vaccines and Infections Research Group (GENVIP), Instituto de Investigación Sanitaria de Santiago, University of Santiago, Santiago de Compostela, Spain; 6 Department of Pediatrics, Translational Pediatrics and Infectious Diseases, Hospital Clínico Universitario de Santiago de Compostela, Santiago de Compostela, Spain; 7 Centro de Investigación Biomédica en Red de Enfermedades Respiratorias (CIBERES), Madrid, Spain

**Keywords:** Meningococcal infection, Sequelae, Economic evaluation, Systematic review

## Abstract

**Background:**

Invasive meningococcal disease (IMD) is uncommon, life-threatening, with many diverse sequelae. The aims were to: 1) comprehensively characterise the sequelae; 2) have a systematic application for sequelae impact in economic evaluation (EE).

**Methods:**

Sequelae categorised as physical/neurological or psychological/behavioural were identified from a systematic review of IMD observational studies (OS) and EEs in high-income countries (published 2001–2020). A comprehensive map and EE-relevant list, respectively, included physical/neurological sequelae reported in ≥2OS and ≥ 2OS + 2EE (≥1OS and ≥ 1OS + 1EE for psychological/behavioural). Sequelae proportions were selected from the highest quality studies reporting most sequelae. Three medical experts independently evaluated the clinical impact of findings.

**Results:**

Sixty-Six OS and 34 EE reported IMD sequelae. The comprehensive map included 44 sequelae (30 physical/neurological, 14 psychological/behavioural), of which 18 (14 physical/neurological and 4 psychological/behavioural) were EE-relevant. Experts validated the study and identified gaps due to limited evidence, underreporting of psychological/behavioural sequelae in survivors/their families, and occurrence of multiple sequelae in the acute phase and long-term.

**Conclusions:**

The considerable burden of IMD sequelae on survivors and their families is potentially underestimated in EE, due to underreporting and poorly-defined subtle sequelae. When assessing IMD burden and potential interventions e.g., vaccination, sequelae range and duration, underreporting, and indirect burden on dependents should be considered.

**Supplementary Information:**

The online version contains supplementary material available at 10.1186/s12889-022-13342-2.

## Highlights

Invasive meningococcal disease survivors frequently suffer from variable sequelae.

A broad sequelae map plus those relevant to economic evaluation (EE) were defined.

Forty-four sequelae were selected: 30 physical/neurological and 14 psychological/behavioural.

Eighteen EE-relevant sequelae included 14 physical/neurological and 4 psychological/behavioural

Underreporting, family impact and multiple sequelae cases were critical gaps.

A video summary linked to this article can be found on Figshare: 10.6084/m9.figshare.19753840.

**Supplementary Information:**

The online version contains supplementary material available at 10.1186/s12889-022-13342-2.

## Background

Invasive meningococcal disease (IMD) is a life-threatening infectious disease, caused by the gram-negative bacterium *Neisseria meningitidis (N. meningitidis)* [1]*.* Disease incidence is highest in infants and young children, followed by adolescents i.e., 8.2 microbiologically-confirmed cases per 100,000 population in infants under 1 year of age, 2.5 in children aged 1–4 years, and 1.0 in adolescents aged 15–24 years across the European Union/European Economic Area in 2016 [2–4]. Overall incidence rates are considerably lower in the United States (US) (overall 0.13/100,000 in 2017), yet they follow similar age-related patterns [5].

IMD has a fluctuating epidemiology with variable *N. meningitidis* serogroup distribution worldwide [6, 7]. Six *N. meningitidis* serogroups (A, B, C, W, X and Y) are responsible for most IMD cases worldwide [5]. Serogroup B is currently the most prevalent in Europe and the US [2, 3], but there are increasing reports of serogroup W cases from Europe, South America and Australia [7]. IMD can be prevented with available vaccines targeting serogroups C, ACWY or B. The national immunisation programs of different countries, however, include different vaccines for IMD prevention. Following the introduction of meningococcal conjugate C vaccines in 1999 (and later quadrivalent meningococcal ACWY vaccines), initially in the United Kingdom (UK), the incidence of IMD due to serogroup C declined in both vaccinated and unvaccinated populations, due to herd immunity [8]. The adoption of meningococcal B vaccine (4CMenB) into the national immunisation programs in the UK and regions of Italy has led to further important reductions in IMD incidence caused by serogroup B [9, 10] and W IMD (using modelling to estimate the direct impact of 4CMenB, based on real-world serogroup W data, pre- and post-vaccination) [11]. A retrospective cohort study in the UK of IMD cases diagnosed between 2008 and 2017 shows a decrease in IMD in young children 0–4 years of age and in adolescents reflecting the introduction of 4CMenB infant vaccination in 2015 and adolescent quadrivalent ACWY vaccination in 2016 [12].

Common clinical presentations of IMD include meningitis and septicaemia, which can lead to high case fatalities in the acute phase, as well as permanent, and sometimes very severe, sequelae in IMD survivors [13]. The long-term sequelae of IMD significantly impact the health of patients as well as their families and social contacts in general. However, the IMD burden beyond the individual patient is poorly defined [14].

It is important to comprehensively assess the impact of the disease burden when evaluating interventions to prevent or treat IMD [12, 13]. Several gaps in the literature have been identified [14] e.g., studies in IMD survivors may not always capture sequelae that take time to develop, due to insufficient follow-up. Because IMD is uncommon and can produce a wide range of sequelae, observational studies (OS) typically contain small numbers of patients and report different sequelae, and few are case-control studies. Economic evaluation (EE) in IMD is often used to inform policy decisions regarding new preventative or therapeutic interventions. It is, therefore, crucial to systematically identify, define, and evaluate all potential IMD sequelae to understand the full disease burden and to define the clinical burden of interest to policy decision-makers.

The objectives of this study were 1) to comprehensively map the broad range of IMD sequelae with respect to manifestation, severity and duration, via systematic literature review (SLR), and 2) to provide a systematic approach to identify the clinical impact of sequalae of relevance to EE to inform policy decision-making. A video summary linked to this article can be found on Figshare: 10.6084/m9.figshare.19753840.

## Methods

### Data collection

An SLR was undertaken to identify both OS and EE in IMD published between 2001 and 2020. Medline, Embase and the Cochrane library (for economic studies) were searched, combining disease terms and outcome terms for sequelae and quality of life (QoL) in the OS search strategy, with economic terms in the EE search strategy. The OS search was an update of a previous SLR [14] and included publications in English, French, German, Dutch and Spanish from high-income countries (as defined by the Organisation for Economic Cooperation and Development) from 1 August 2001 to 2 April 2020 (Table S1.1A). The EE systematic search was an update of a previous comprehensive literature review conducted as part of an EE [15], and included publications in English and French from 1 August 2001 to 2 June 2020 (Table S1.1B). Titles and abstracts were screened by two independent reviewers (KL & MN), using pre-defined inclusion and exclusion criteria (based on PICOS in Table S1.2 i.e., patients, interventions, comparisons, outcomes and study designs of interest). Selected full-text OS articles were critically analysed using the Scottish Intercollegiate Guidelines Network (SIGN) framework, in which the level of evidence is graded from ‘1++’ (i.e., high-quality meta-analysis, systematic review of clinical trials) to 4 (i.e., expert opinion). The methodological quality of included full-text EE articles was assessed by the Philips et al. (2004) checklist [16]. Data were extracted into pre-defined data extraction tables: author, year, country, study design, population, outcomes, study period, disease onset characteristics, serogroups and results. The PRISMA flow diagrams (Figs. S1.2 and S1.3) and checklist (Table S1.3) are presented in Supplementary file 1.

### Data analysis

**Fig. 1:**
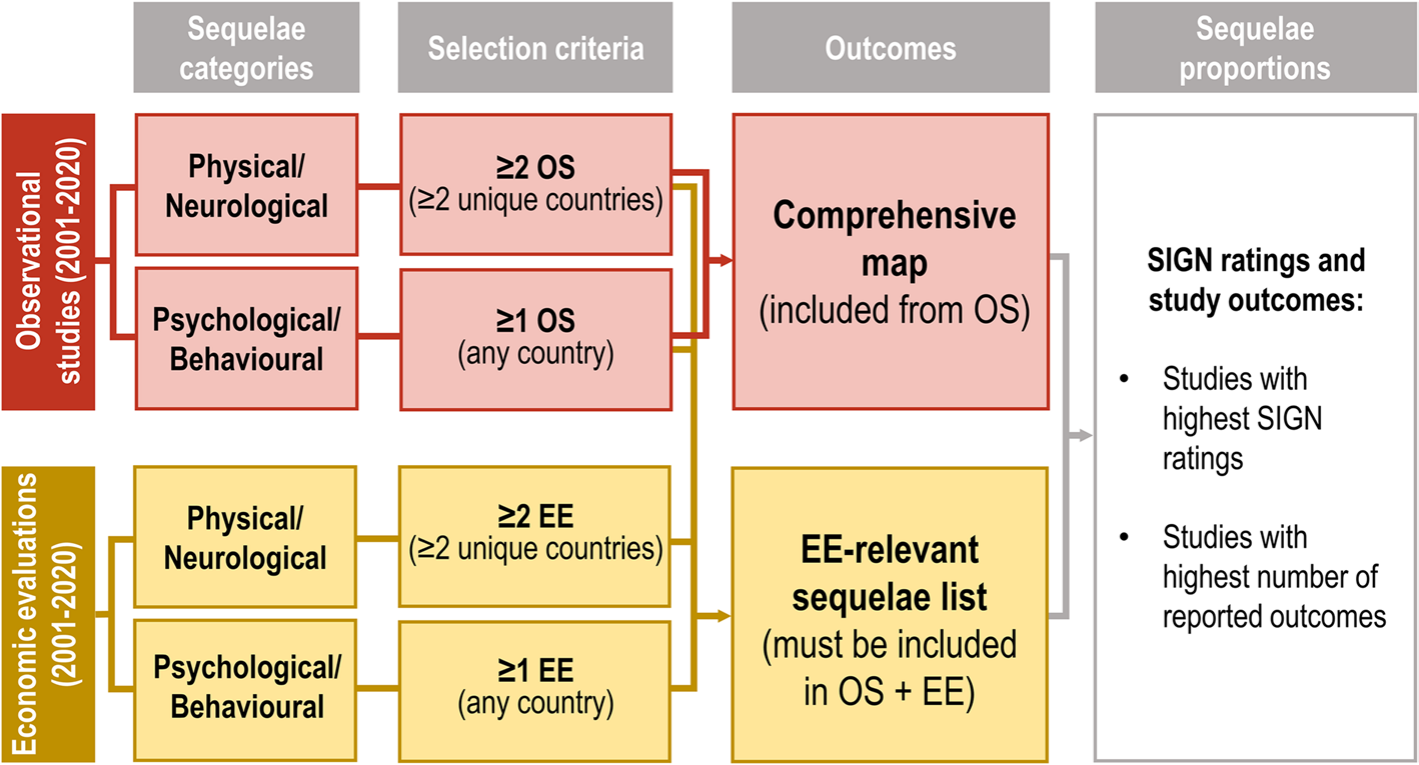
Sequelae selection criteria for comprehensive map (from OS) and EE-relevant list (from OS+EE), with sequela proportions from studies with highest SIGN rating and number of outcomes. EE: economic evaluation; OS: observational study; SIGN: Scottish Intercollegiate Guidelines Network**.** Sequelae selected for the comprehensive map must be reported in at least 2 observational studies (OS) for physical/neurological sequelae and at least 1 OS for psychological/behavioural sequelae. Sequelae proportions were taken from highest SIGN-rated studies reporting the greatest number of sequelae. Sequelae from this map relevant to economic evaluation (EE) must also be reported in at least 2 health economic studies (HES) for physical/neurological sequelae and at least 1 HES for psychological/behavioural sequelae

Three infectious disease experts participated in the key analysis steps, using a systematic procedure to independently review and validate the methods with respect to their clinical validity. The following methodological steps were undertaken and are described in more detail below: a) definition of a list of possible IMD sequelae to which reported sequelae can be mapped (Table S2.1), b) pre-define criteria for the objective selection of sequelae (criteria presented in Fig. 1) for the comprehensive map (Table S2.2 and Table S2.3) and the EE-relevant list (Table S2.4), c) categorisation of sequelae as physical/neurological or psychological/behavioural, and d) pre-defined criteria for the objective selection of sequela probabilities from studies.

As there has been no conclusive evidence to suggest IMD sequelae differ by serogroup [14], all potential IMD sequelae were compiled, irrespective of serogroup. The previous SLR [14] found that sequelae reported in the literature may be reported using different terminology. In order to be concise and consistent, the sequelae identified in the previous SLR were used to support and define the list of all potential IMD sequelae presented in this study, with assumptions about which conditions are included within each definition (see Supplementary file 2, Table S2.1). Based on the data extraction, sequelae reported in the literature were then mapped to these definitions and categorised as physical/neurological or psychological/behavioural.

Criteria for selecting IMD sequelae, for both the comprehensive map and list of EE-relevant sequelae, were validated by all experts. The comprehensive map included sequelae reported in OS only. Physical/neurological sequelae were selected if the condition was reported in at least two OS from two unique countries. For psychological/behavioural sequelae, which were expected to be underreported, the requirement for selection was for conditions to be reported in at least one OS (Fig. 1). To identify sequelae from the comprehensive map that are relevant for EE, physical/neurological sequelae must also be reported in at least two EE and psychological/behavioural sequelae in at least one EE (Fig. 1).

As multiple studies could have reported the probability of a given sequelae, the final proportion of cases with a sequela was selected among the studies with the highest SIGN ratings, from which the study with the highest number of reported sequelae was used (Fig. 1). The range of reported values across these top-rated studies for a given sequela is also presented (see Supplementary file 2 for data extraction by sequela [Table S2.2]).

## Results

### Overview of identified studies

**Fig. 2:**
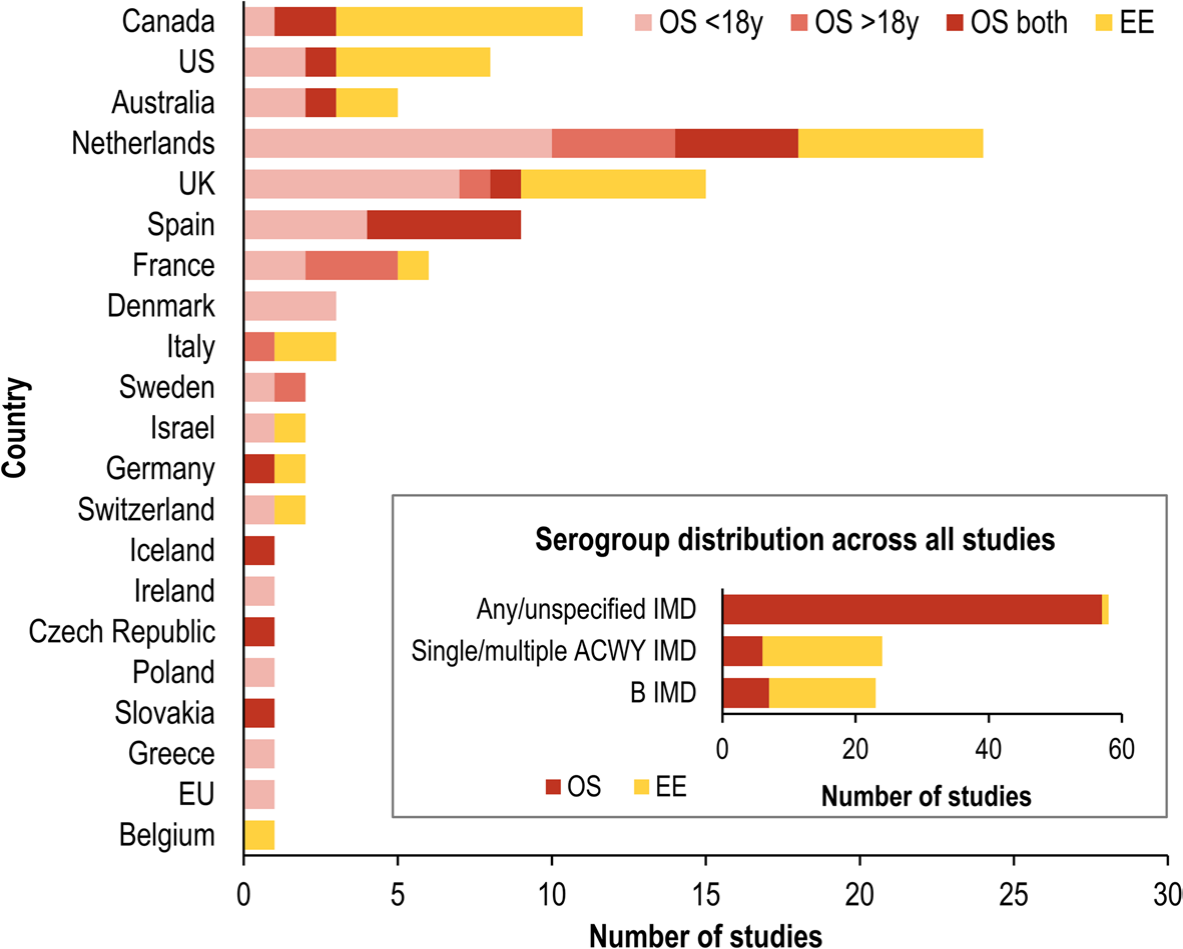
Distribution by geographic location of OS (with age at IMD diagnosis) and EE, and by IMD serogroup. ACWY/B IMD: single or multiple serogroup(s) A,C,W,Y or B invasive meningococcal disease; EE: economic evaluation; EU European Union (i.e., Austria, Germany, Lithuania, Netherlands, Spain, Switzerland, UK); OS: observational study; UK United Kingdom; US United States; SIGN: Scottish Intercollegiate Guidelines Network. Sequelae selection criteria for comprehensive map (from OS) and EE-relevant list (from OS+EE), with sequela proportions from studies with highest SIGN rating and number of outcomes. The distribution of the 66 observational studies (OS) and 34 health economic studies (HES) by country (if > 2 studies conducted per country), and by serogroup

The SLR identified 66 OS and 34 EE providing relevant information on IMD sequelae in survivors across 22 countries. The majority of OS reported outcomes for IMD of any serogroup (non-specified), while the majority of EE focused on serogroup-specific IMD (i.e., serogroup B or single/multiple A,C,W,Y serogroups) for interventions targeting IMD caused by these serogroups (Fig. 2). In terms of geographic location, the OS were largely conducted in the Netherlands, UK and Spain, while countries with more than three EEs were Canada, the US, UK and Netherlands (Fig. 2). Overall, 38 OS were conducted in children (< 18 years), 10 in adults and 17 in both (1 unspecified [17]) (Fig. 2). The duration of follow up in OS ranged from initial hospitalisation to 30 years post-diagnosis. The EE were typically lifetime economic models that assessed patients from birth until death.

**Fig. 3:**
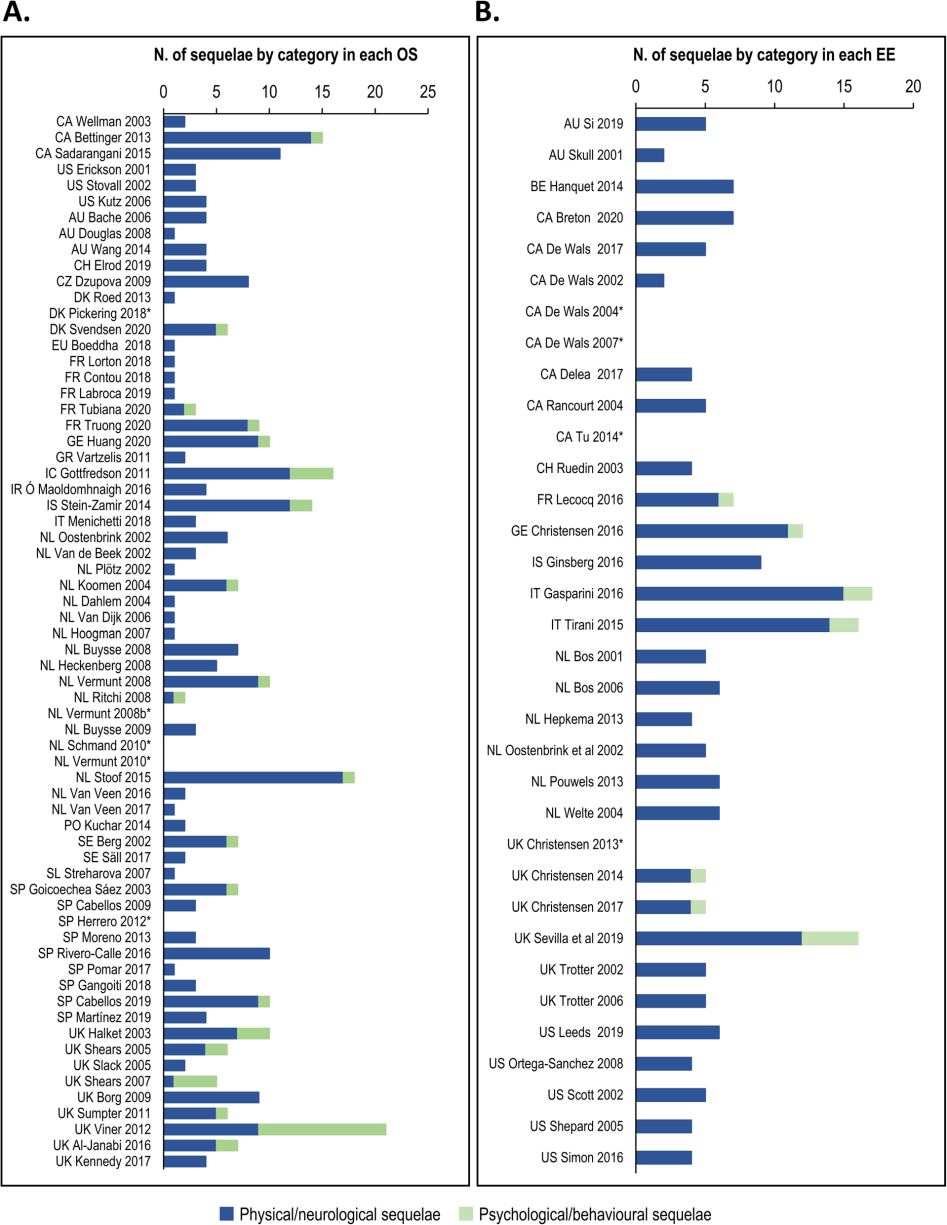
Number of IMD sequelae by category reported in **A**) each OS and **B**) each EE. AU: Australia; BE: Belgium; CA: Canada; CH: Switzerland; CZ: Czech Republic; DK: Denmark; EE: economic evaluation; EU: European Union; FR: France; GE: Germany; GR: Greece; IC: Iceland; IMD: invasive meningococcal disease; IR: Ireland; IS: Israel; IT: Italy; N.: number; NL: Netherlands; OS: observational study; PO: Poland; SE: Sweden; SL: Slovakia; SP: Spain; UK: United Kingdom; US: United States (of America) *Note: studies reporting number but not type of sequelae or grouping sequelae into a composite outcome. The number of sequelae reported per observational study (OS) and per health economic study (HES) categorised as physical, neurological or psychological/behavioural

Among the 66 OS, the majority reported physical/neurological (92%) sequelae and 30% reported psychological/behavioural sequelae. Both categories of sequelae were observed in IMD cases and reported in studies from 2002 onwards. A small number of OS studies reported 15 or more sequelae e.g., Gottfredsson et al (2011) [18], Viner et al. (2012) [19], Bettinger et al. (2013) [20] and Stoof et al. (2015) [21] (Fig. 3a). Among the 34 EE, most reported physical/neurological (88%) sequelae and 21% reported psychological/behavioural sequelae. Prior to 2015, EE included fewer IMD sequelae. From 2014 onwards, some EE began including psychological/behavioural sequelae. Two EE in Italy and one in the UK included more than 15 sequelae i.e., Gaspirini et al. (2016) [22], Tirani et al. (2015) [23] and Sevilla et al. (2019) [24] (Fig. 3b).

**Fig. 4:**
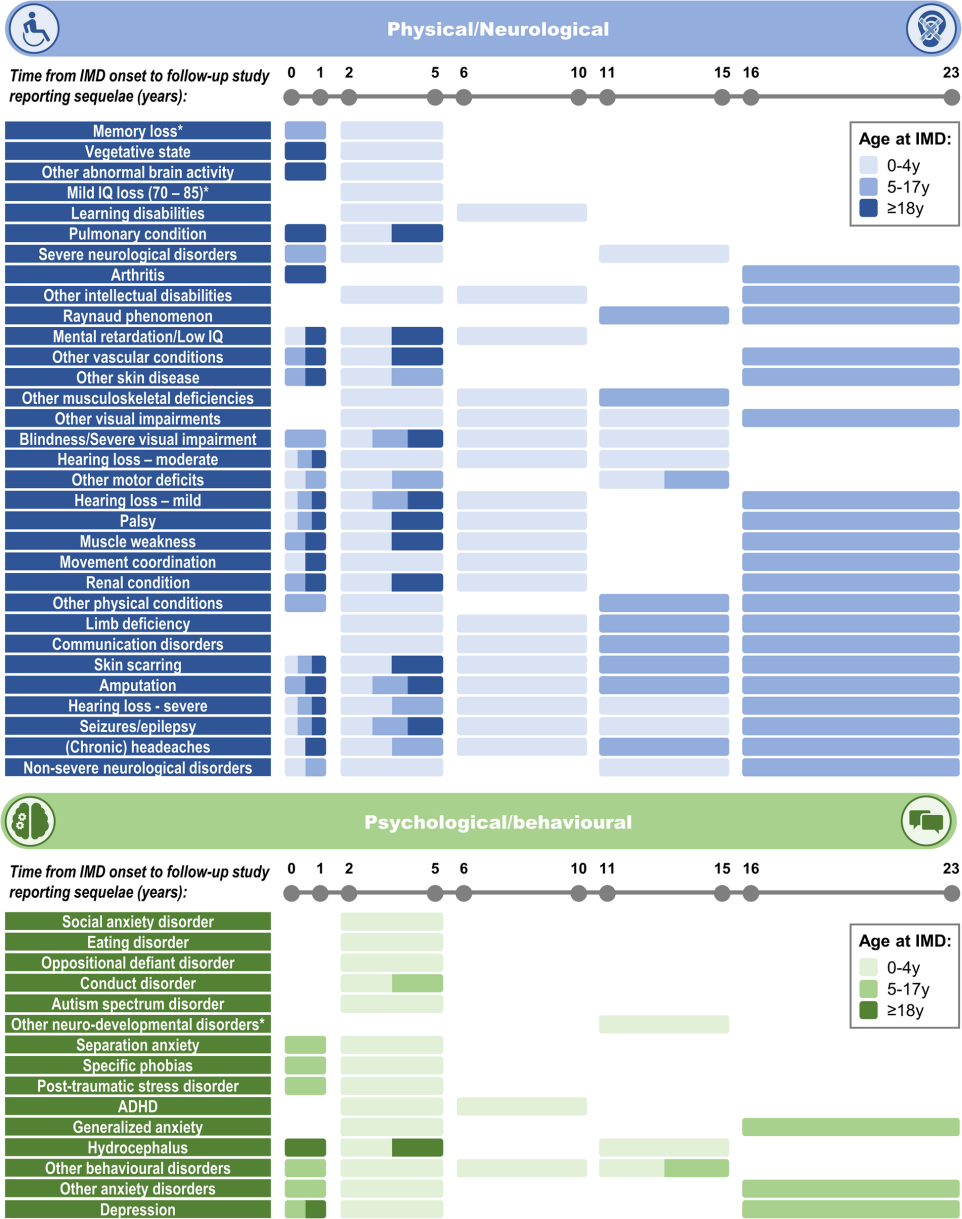
Time from IMD onset to follow-up study reporting each physical/neurological and psychological/behavioural sequela, by age group at IMD onset. The time from IMD onset (for age groups 0–4 years, 5–17 years and 18+ years) to follow-up study reporting each physical/neurological and psychological/behavioural sequela is presented

To better understand the potential duration of sequelae, Fig. 4 presents an overview of time from IMD onset to follow-up study reporting each sequela, for IMD cases aged 0–4 years, 5-17y and 18+ years. Many physical/neurological sequelae were reported in studies with long follow-up durations of up to 16–23 years, and sequelae such as hearing loss, seizures and skin scarring were often investigated in follow-up studies in all age groups. The majority of psychological/behavioural sequelae, however, had a maximum follow-up duration of 2–5 years post-diagnosis, were mostly reported by Viner et al. (2012) [19], and most data were from IMD cases occurring in infants and young children aged 0–4 years (Fig. 4).

### Comprehensive map of IMD sequelae

The range of possible IMD sequelae from the previous SLR, to which the sequelae identified in this study were mapped, is presented in Table S2.1.

The sequelae selection criteria and resulting sequelae list was validated independently by the clinical experts. The combined process led to the selection of 44 IMD sequelae in the comprehensive map; 30 physical/neurological and 14 psychological/behavioural sequelae (Table 1).

**Table 1:** Comprehensive IMD sequelae map: selected sequelae proportions (range across studies), and relevance for economic evaluation

Category / clinical presentation	Sequelae % (range across studies)	Relevant for EE	Source SIGN
**Category: Physical/neurological sequelae**			
**Renal condition**	7.32 (0.79–8.92)	Yes	Huang 2020 [25] 2+
**Hearing loss – unilateral/hearing impairment**	6.47 (2.30–12.94)	Yes	Viner 2012 [19] 2++
**Non-severe neurological disorders**	5.02 (1.26–12.17)	Yes	Rivero-Calle 2016 [26] 2+
**Hearing loss – moderate bilateral**	4.74 (3.32–4.74)	Yes	Viner 2012 [19] 2++
**Communication disorders**	4.18 (0.39–12.17)	Yes	Viner 2012 [19] 2++
**Motor deficit - composite of:**	3.97	Yes	
** Muscle weakness**	2.47 (0.26–5.83)		Cabellos 2019 [27] 2+
** Palsy**	0.26 (0.26–7.93)		Bettinger 2013 [20] 3
** Movement coordination/ balance deficits**	0.22 (0.00–16.83)		Rivero-Calle 2016 [26] 2+
** Other and non-specific motor deficits**	1.02 (1.02–12.17)		Bettinger 2013 [20] 3
**Skin scarring (with/without grafting)**	3.66 (1.67–17.82)	Yes	Huang 2020 [25] 2+
**Hearing loss – severe/profound bilateral (cochlear implant)**	2.45 (1.53–11.88)	Yes	Viner 2012 [19] 2++
**Seizures/epilepsy**	2.09 (0.92–7.06)	Yes	Viner 2012 [19] 2++
**Other and non-specified skin disease (e.g., skin necrosis, eczema, psoriasis)**	1.53 (1.53–2.40)	Yes	Rivero-Calle 2016 [26] 2+
**Amputation**	1.26 (major) 2.09 (major and minor) (1.74–3.84)	Yes	Viner 2012 [19] 2++
**Severe neurological disorders**	1.02 (1.02–1.18)	Yes	Bettinger 2013 [20] 3
**Mental retardation/low IQ**	0.84 (0.52–2.44)	Yes	Viner 2012 [19] 2++
**Blindness/severe visual impairment**	0.42 (0.26–2.44)	Yes	Viner 2012 [19] 2++
Raynaud phenomenon symptoms	27.72		Borg 2009 [28] 2+
Other and non-specified vascular conditions	15.24 (0.26–15.24)		Huang 2020 [25] 2+
Chronic headaches	13.91 (13.91–18.33)		Stein-Zamir 2014 [29] 3
Other and non-specific physical conditions	12.87 (0.92–12.87)		Borg 2009 [28] 2+
Learning disabilities*	11.76		Svendsen 2020 [30] 2+
Pulmonary condition	10.82 (0.13–10.82)		Cabellos 2019 [27] 2+
Arthritis	5.73 (5.73–7.59)		Gottfredson 2011 [18] 2-
Limb deficiency/deformities	3.96 (3.96–8.70)		Borg 2009 [28] 2+
Other and non-specific intellectual disabilities	1.48 (0.63–22.61)		Gottfredson 2011 [18] 2-
Other visual impairments	1.13 (0.92–1.13)		Sadarangani 2015 [31] 2-
Other and non-specified musculoskeletal deficiencies	0.44 (0.44–3.65)		Rivero-Calle 2016 [26] 2+
Other and non-specified abnormal brain activity	0.26		Bettinger 2013 [20] 3
Vegetative state	0.13		Stoof 2015 [21] 3
**Category: Psychological/behavioural sequelae**			
**ADHD**	11.41	Yes	Viner 2012 [19] 2++
**Separation anxiety**	6.85	Yes	Viner 2012 [19] 2++
**Generalised anxiety**	2.68 (2.68–5.83)	Yes	Viner 2012 [19] 2++
**Depression**	0.26 (0.00–5.83)	Yes	Bettinger 2013 [20] 3
Oppositional defiant disorder	11.41		Viner 2012 [19] 2++
Other and non-specific anxiety disorders	6.67 (0.00–6.67)		Gottfredson 2011 [18] 2-
Conduct disorder	6.04		Viner 2012 [19] 2++
Specific phobias	4.70		Viner 2012 [19] 2++
Other and non-specific emotional/behavioural disorders	3.41		Stoof 2015 [21] 3
Hydrocephalus	2.44		Huang 2020 [25] 2+
Autistic spectrum disorder	1.34		Viner 2012 [19] 2++
Social anxiety disorder/social phobia	1.34		Viner 2012 [19] 2++
Eating disorder	0.68		Viner 2012 [19] 2++
Post-traumatic stress disorder	0.00		Viner 2012 [19] 2++

*Note:* No range is reported when sequela was from a single study

*ADHD* Attention-deficit/hyperactivity disorder, *EE* Economic evaluation, *IMD* Invasive meningococcal disease, *IQ* Intelligence quotient, *SIGN* Scottish Intercollegiate Guidelines Network

^a^Aggregate % reported for: learning disabilities, behavioural problems and memory loss, used as a proxy for ‘learning disabilities’

There was a wide range of reported proportions of cases with sequelae across included studies. The selected proportion for each sequela was chosen from the highest quality studies reporting the greatest number of sequelae (Table 1). The sum of all IMD sequelae proportions in Table 1 is over 40%. While some studies reported more types of sequelae than others, the broad range of IMD sequelae was not captured from a single study but across multiple studies i.e., 11 OS reporting physical/neurological sequelae, and 5 OS reporting psychological/behavioural sequelae. Although IMD is not commonly seen in clinical practice, some reported sequelae proportions were found to be either lower than experts’ expectations (e.g., skin scarring, limb deficiencies/deformities, other and non-specified musculoskeletal deficiencies) or higher (e.g., Raynaud phenomenon, other and non-specified vascular conditions and hydrocephalus). However, the high proportion of IMD cases with some sequelae, such as Raynaud phenomenon, was deemed plausible, as the specific study (Borg et al. 2009 [28]) was designed to capture this outcome (Table S2.3).

### IMD sequelae relevant for EE

Among the sequelae in the comprehensive map, 18 sequelae met the criteria and were deemed more relevant for EE based on previous inclusion in economic studies; these included 14/30 physical/neurological sequelae and 4/14 psychological/behavioural sequelae. The physical/neurological sequelae were captured by five different studies and psychological/behavioural from two studies, with wide variations in reported proportions with sequelae across studies (Table 1). The sequelae selected for EE were qualified as relevant from the clinical perspective. It is likely that sequelae not widely reported in OS were also not included in previous EEs. As the EE list was based on sequelae from previous EEs, there may be relevant sequelae from the comprehensive map that have never been considered in EE e.g., learning disabilities, pulmonary conditions and susceptibility to respiratory illnesses (like respiratory syncytial virus and influenza) can impact economic burden (Table S2.4).

Supplementary file 2 contains the sequelae data extraction and expert comments on mapping reported outcomes (Table S2.1), sequela selection methodology (Table S2.2), sequela proportions selected (Table S2.3), and EE-relevant sequelae (Table S2.4).

## Discussion

This study comprehensively analysed observational and economic studies from 2001 to 2020, to determine the clinically-relevant burden of IMD sequelae. A particular focus was on the sequelae included in EE. Using a systematic approach, 44 sequelae of varying severity were included in the comprehensive map, of which 18 were classed as important for EE. The physical/neurological and psychological/behavioural sequelae were caused by any serogroup, and some were permanent and disabling.

With the exception of 6/66 OS and 4/34 EE that reported over 10 IMD sequelae, most other studies reported a narrow range of sequelae, with on average only one psychological/behavioural sequela (Fig. 3). It is important to note that physical/neurological sequelae are often apparent e.g., amputation, skin scarring or hearing loss, and are, thus, likely to be reported. In contrast, certain psychological/behavioural sequelae are more insidious, may take time to become apparent, or may not be attributed to IMD, and thus may evade reporting. In a recent retrospective case-control study of IMD in the UK, psychological sequelae were reported after a median of 15.5 months (up to 36.2 months in infants) while neurological sequelae were reported after a median of 8.5 months and physical sequelae, a median of 1 month [12]. As such, the risk of under-reporting and under-estimation of IMD burden due to psychological/behavioural sequelae is high. The design of OS intended to capture the full extent of psychological/behavioural sequelae of variable severity and duration is demanding, since extended observation periods are required, as are sophisticated designs including blinded investigators, adequate control groups, tools to determine coping mechanisms [32], and so forth.

EEs tend to focus on quality-adjusted life-years and cost impact in cost-effectiveness analysis, therefore, sequelae that have an important adverse effect on health-related quality of life (HRQOL) or survival, and those with long-term or high costs, are particularly relevant for EE. EEs with a more comprehensive approach e.g., those including a wide range of sequelae with their associated impact on HRQOL of patients and caregivers as well as long-term costs [22–24] are better able to depict the cost-effectiveness of IMD interventions, an essential factor when used to guide policy decisions [33]. By contrast, EEs that include a narrow range of sequelae may underestimate the burden of IMD and its health and economic impact, and thus fail to grasp the cost-effectiveness of IMD interventions, as demonstrated in Beck et al. (2021) [15]. A recent systematic review of economic evaluations in depression has shown that changing from a narrow payer perspective to a broader societal perspective (e.g., considering the burden on caregivers as well as patients) can change the outcome of the analysis and thus affect policy decisions [34]. Systematic reviews are better suited to capture the burden of disease and thus to be used in EEs, yet they rely on the quality of included studies.

The evaluation of study findings from a clinical and real-world perspective highlighted several gaps and areas for future research. Firstly, regarding the list of possible sequelae, several factors can affect whether sequelae are reported in relation to IMD. For example, some sequelae that receive high attention are rare in IMD survivors e.g., palsy, non-specified disruptive impulse control and conduct disorders, vegetative state, and substantial post-traumatic stress disorders (PTSD). Moreover, age substantially impacts the diagnosis of sequelae e.g., PTSD and depression may be easier to diagnose in adults than in young children. Some sequelae may be attributed to other medical conditions or may vary greatly in severity, resulting in a variable disease impact e.g., other/non-specific anxiety disorders, conduct disorder, autism, depression and oppositional defiant disorder. A number of sequelae are not typically assessed in studies, including pain, dysesthesia and aesthetic sequelae related to skin scarring.

In view of the comprehensive map, the impact of IMD on HRQOL, on other household members, survivor dependency and potentially important financial consequences where productivity of patients and caregivers is impacted [14] was usually not adequately captured in studies assessing psychological sequelae. Thus, underreporting of psychological/behavioural sequelae concerns both patients and family members. As such there is a discrepancy in the depiction of these sequelae in the literature versus clinical experience. The questions raised highlight the scarcity of available evidence in IMD survivors and the need to conduct further research to better grasp the lifelong impact of sequelae on patients and family members.

A wide range of sequela proportions were reported across studies in some cases (Table S2.2), even among high-rated studies (defined by high SIGN quality rating and high number of reported sequelae) (Table S2.3). Better strategies are, therefore, needed to select and justify the chosen point estimates and reduce uncertainty resulting from highly variable sequelae incidence. The strategy selected in this analysis prioritised high-quality studies with the largest number of sequelae outcomes reported. Robust estimates of sequela proportions may be best achieved by increasing the sample sizes, although this remains a major challenge for uncommon diseases such as IMD. In this analysis, it was not possible to pool data due to heterogeneity across study populations and designs. Future analyses may give more weight to findings from controlled studies, such as the case-control study by Viner et al. (2012) [19] and Guedes et al. (2021) [12]. Many studies lacked healthy control groups, which made interpretation of subtle differences in neurological sequelae difficult e.g., an intelligence quotient (IQ) loss below 10 points is likely to evade analysis in many studies.

Finally, regarding the EE-relevant list of sequelae, more specific definitions should be considered for neurological and neuropsychological disorders including depression. For example, the term depression covers a broad range of disease severity with variable, and often ill-defined, economic impact, in particular if it occurs with other sequelae. The sequelae considered for EE represent an important burden for IMD survivors, with clinical observations suggesting a potentially significant proportion of patients suffer from these conditions likely to lead to higher medical costs and require long-term care. The clinical evaluation also identified EE-relevant sequalae that were not included in previous EE, but which may have high or long-term costs or an important HRQOL impact, e.g., pulmonary conditions and learning disabilities.

In clinical practice, multiple organs are found to be affected both in the acute phase of IMD or over the lifetime of some survivors. The numbers of IMD patients with multiple sequelae seems, however, to be inadequately reported or quantified in OS. In a study by Darton et al. (2009) [35], nearly 30% of patients with IMD were reported to have physical and/or neurological sequelae, based on a limited list of sequelae considered i.e., defined as the need for dialysis, limb loss, skin graft, need for audiometry or visual impairment. In a US managed care population, Karve et al. (2011) [36] identified 34% of IMD patients with sequelae based on a list of up to 15 possible sequelae investigated, whereas Davis et al. (2011) [37] identified 41% of IMD patients with sequelae investigating up to 18 possible physical/neurological sequelae [4]. These studies did not consider the full range of physical/neurological and psychological/behavioural sequelae of IMD. Based on our analysis, the sum of all sequelae proportions is over 40%, but given the scarce evidence and underreporting, it is difficult to speculate if this number represents the proportion of survivors with at least one sequela, however, this does suggest a potentially high prevalence of sequelae in survivors. This is supported by the recent UK case-control study which found that 43.8% of IMD cases developed at least one sequela, compared with 23.1% of controls [12]. Further investigations should focus on the prevalence of multiple sequelae and how to address their combined impact in EE. The sequelae proportions also need further investigation due to the limited evidence available and under-reporting of psychological sequelae.

This study has a number of limitations. Comparing results across studies was challenging due to variations in study design, disease manifestation, age of subjects, study population investigated, time points and length of follow-up, as well as definitions and types of assessment used for sequelae. This heterogeneity resulted in a wide range of reported sequelae proportions and made pooling of data difficult. A more sophisticated approach is needed in future for separate analysis of data in children and adults e.g., where countries may consider different age groups for vaccination. As observed in the UK, vaccination of children and adolescents has reduced IMD incidence in these age groups, but adults are still affected, with significantly higher risks versus controls of mortality, driven by cases over 25 years old, and of severe sequelae, by cases over 50 years old [12]. The sequelae reported in each study varied considerably. Thus, additional criteria based on SIGN study quality rating and number of outcomes per study were considered, however there is still room for bias in the outcomes reported. Further statistical analysis should consider either applying standardisation methods (matching propensity analysis) to correct for patient characteristics in the study populations, or bootstrapping approaches to confirm sequelae trends where data is limited (although this alone may impose many challenges) or should focus on case-control studies. Additionally, to better understand the natural history of the disease, studies may consider analysing and applying time to follow up data to track sequelae development or confirm sequelae distribution over patients’ lifetime, or conduct sub-group analysis to determine potential risk factors (e.g., age [12]). Some generic definitions were used to group non-specific sequelae in this analysis, as not all studies clearly defined specific sequelae.

The comprehensive list of sequelae, including assumptions of which conditions are included in each definition, may be a helpful classification to harmonise reporting in future studies and allow for comparisons across studies. This study has highlighted the importance of not underestimating the burden of psychological sequelae on patients and caregivers. During the COVID-19 pandemic, caregivers experienced significantly more mental health issues than non-caregivers, highlighting some of the difficulties families face in managing chronic conditions [38]. This should be accounted for in future studies, especially when considering multifactorial disease burden i.e., COVID-19 in IMD survivors could increase the spillover burden on caregivers. While data on co-infection of IMD and COVID-19 are rare [39], a recent study in the UK in patients with invasive pneumococcal disease (IPD) co-infected with COVID-19 found the risk of death increased significantly in co-infected patients compared to IPD or COVID-19 alone [40]. During the pandemic, there was a significant decline in invasive diseases transmitted by respiratory droplets, including IMD, likely as a result of containment measures put in place to prevent SARS-CoV-2 transmission. Several of these deadly invasive diseases are vaccine-preventable, and continued vaccination can help prevent a potential increase in burden once containment measures are lifted [41, 42]. The restrictions also resulted in disruptions to vaccination provision, including childhood vaccination for IMD in some countries [41]. UK surveillance data has shown an increase in serogroup B IMD cases in the months following easing of social distancing measures, and primarily among adolescents, who are not vaccinated with 4CMenB [43].

## Conclusion

This study comprehensively reviewed existing literature with a clinical evaluation of IMD sequelae. The results demonstrate the considerable burden that IMD survivors may experience due to physical/neurological and psychological/behavioural sequelae, and the need for additional research to quantify the impact. The full sequelae impact has been previously underestimated in EE, due to challenges in synthesising scarce evidence, and underreporting of psychological/behavioural sequelae. Comprehensive consideration of these factors (e.g., underreported sequelae, multiple sequelae, burden beyond the patient) is warranted when assessing IMD burden and potential interventions to reduce it, such as vaccination programs.

## Supplementary Information

**Additional file 1: Supplementary File 1.** Systematic literature review. **Fig. S1.1.** Plain Language Summary. **Table S1.1.** Search strategies (A. Observational / B. Health economic)**. Table S1.2.** PICOS inclusion/exclusion selection criteria. **Fig. S1.2.** PRISMA flow diagram – observational study searches. **Fig. S1.3.** PRISMA flow diagram – health economic study searches. **Table S1.3.** Completed 2009 PRISMA checklist.

**Additional file 2: Supplementary File 2.** Data extraction and expert validation. **Table S2.1.** Pre-defined list of IMD sequelae with definitions, to which reported sequelae are mapped – expert validation comments. **Table S2.2.** Sequelae data extraction from high-rated studies (highest SIGN rating and highest number of sequelae reported). **Table S2.3.** IMD sequelae for comprehensive map: selected percentage (range across studies) – expert validation comments. **Table S2.4.** IMD sequelae relevant for economic evaluation: selected percentage (range across studies) – expert validation comments.

## Data Availability

All data generated or analysed during this study are included in this published article [and its supplementary information files].

## Abbreviations

EE: Economic evaluation
HRQOL: Health-related quality of life
IMD: Invasive meningococcal disease
IQ: Intelligence quotient
*N. meningitidis*: *Neisseria meningitidis*
OS: Observational studies
PICOS: Patients, Interventions, Comparisons, Outcomes and Study designs
PTSD: Post-traumatic stress disorders
QoL: Quality of life
SIGN: Scottish Intercollegiate Guidelines Network
SLR: Systematic literature review
UK: United Kingdom
US: United States
4CMenB: 4-component meningococcal B vaccine

## References

[1] National Organization for Rare Disorders (NORD). Meningococcal Meningitis. https://rarediseases.org/rare-diseases/meningococcal-meningitis/. Accessed 30 June 2021.

[2] Centers for Disease Control and Prevention (CDC). Meningococcal Disease - Surveillance 2019. https://www.cdc.gov/meningococcal/surveillance/index.html. Accessed 30 June2021.

[3] European Centre for Disease Prevention and Control (ECDC). Factsheet about meningococcal disease 2019. https://www.ecdc.europa.eu/en/meningococcal-disease/factsheet. Accessed 30 June 2021.

[4] European Centre for Disease Prevention and Control (ECDC). Surveillance report. Invasive meningococcal disease - Annual epidemiological report for 2017. 2017. https://www.ecdc.europa.eu/sites/default/files/documents/AER_for_2017-invasive-meningococcal-disease.pdf. Accessed 27 July 2021.

[5] Parikh SR, Campbell H, Bettinger JA, Harrison LH, Marshall HS, Martinon-Torres F (2020). The everchanging epidemiology of meningococcal disease worldwide and the potential for prevention through vaccination. J Inf Secur.

[6] World Health Organization (WHO). Serogroup distribution of invasive meningococcal disease, 2019. 2020. https://www.who.int/health-topics/meningitis#tab=tab_1. Accessed 30 June 2021.

[7] Booy R, Gentile A, Nissen M, Whelan J, Abitbol V (2019). Recent changes in the epidemiology of Neisseria meningitidis serogroup W across the world, current vaccination policy choices and possible future strategies. Hum Vaccin Immunother.

[8] Tin Tin Htar M, Jackson S, Balmer P, Serra LC, Vyse A, Slack M (2020). Systematic literature review of the impact and effectiveness of monovalent meningococcal C conjugated vaccines when used in routine immunization programs. BMC Public Health.

[9] Ladhani SN, Andrews N, Parikh SR, Campbell H, White J, Edelstein M (2020). Vaccination of infants with meningococcal group B vaccine (4CMenB) in England. N Engl J Med.

[10] Azzari C, Moriondo M, Nieddu F, Guarnieri V, Lodi L, Canessa C, et al. Effectiveness and impact of the 4CMenB vaccine against group B meningococcal disease in two Italian regions using different vaccination schedules: a five-year retrospective observational study (2014-2018). Vaccines. 2020;8(3). 10.3390/vaccines8030469. 10.3390/vaccines8030469 PMC7563708 32842669

[11] Ladhani SN, Campbell H, Andrews N, Parikh SR, White J, Edelstein M, et al. First real world evidence of meningococcal group B vaccine, 4CMenB, protection against meningococcal group W disease; prospective enhanced national surveillance. England. Clin Infect Dis. 2020. 10.1093/cid/ciaa1244. 10.1093/cid/ciaa1244 32845996

[12] Guedes S, Bricout H, Langevin E, Tong S. Bertrand-Gerentes I. BMC Public Health. 2021. 10.21203/rs.3.rs-880229/v1. 10.1186/s12889-022-12933-3 PMC8928586 35296287

[13] Martinón-Torres F (2016). Deciphering the burden of meningococcal disease: conventional and under-recognized elements. J. Adolesc. Health.

[14] Olbrich KJ, Müller D, Schumacher S, Beck E, Meszaros K, Koerber F (2018). Systematic review of invasive meningococcal disease: sequelae and quality of life impact on patients and their caregivers. Infect Dis Ther.

[15] Beck E, Klint J, Neine M, Garcia S, Meszaros K (2021). Cost-effectiveness of 4CMenB infant vaccination in England: a comprehensive valuation considering the broad impact of serogroup B invasive meningococcal disease. Value Health.

[16] Philips Z, Ginnelly L, Sculpher M, Claxton K, Golder S, Riemsma R (2004). Review of guidelines for good practice in decision-analytic modelling in health technology assessment. Health Technol Assess.

[17] Streharova A, Krcmery V, Kisac P, Kalavsky E, Holeckova K, Lesnakova A (2007). Predictors of inferior outcome in community acquired bacterial meningitis. Neuro Endocrinol Lett.

[18] Gottfredsson M, Reynisson IK, Ingvarsson RF, Kristjansdottir H, Nardini MV, Sigurdsson JF (2011). Comparative long-term adverse effects elicited by invasive group B and C meningococcal infections. Clin Infect Dis.

[19] Viner RM, Booy R, Johnson H, Edmunds WJ, Hudson L, Bedford H (2012). Outcomes of invasive meningococcal serogroup B disease in children and adolescents (MOSAIC): a case-control study. Lancet Neurol.

[20] Bettinger JA, Scheifele DW, Le Saux N, Halperin SA, Vaudry W, Tsang R (2013). The disease burden of invasive meningococcal serogroup B disease in Canada. Pediatr Infect Dis J.

[21] Stoof SP, Rodenburg GD, Knol MJ, Rümke LW, Bovenkerk S, Berbers GA (2015). Disease burden of invasive meningococcal disease in the Netherlands between June 1999 and June 2011: a subjective role for serogroup and clonal complex. Clin Infect Dis.

[22] Gasparini R, Landa P, Amicizia D, Icardi G, Ricciardi W, de Waure C (2016). Vaccinating Italian infants with a new multicomponent vaccine (Bexsero(R)) against meningococcal B disease: a cost-effectiveness analysis. Hum Vaccin Immunother.

[23] Tirani M, Meregaglia M, Melegaro A (2015). Health and economic outcomes of introducing the new MenB vaccine (Bexsero) into the Italian routine infant immunisation programme. PLoS One.

[24] Sevilla J, Tortorice D, Kantor D, Bloom DE, Hogea C, Beck E. PIN43 Lifecycle-model-based economic evaluation of infant meningitis B vaccination in the UK. ISPOR; 2019; Copenhagen, Denmark. Value Health. 2019(22):S647.

[25] Huang L, Heuer OD, Janßen S, Häckl D, Schmedt N (2020). Clinical and economic burden of invasive meningococcal disease: evidence from a large German claims database. PLoS One.

[26] Rivero-Calle I, Vilanova-Trillo L, Pardo-Seco J, Salvado LB, Quinteiro LI, Martinon-Torres F (2016). The burden of pediatric invasive meningococcal disease in Spain (2008-2013). Pediatr Infect Dis J.

[27] Cabellos C, Pelegrín I, Benavent E, Gudiol F, Tubau F, Garcia-Somoza D (2019). Invasive meningococcal disease: what we should know, before it comes back. Open forum. Infect Dis.

[28] Borg J, Christie D, Coen PG, Booy R, Viner RM (2009). Outcomes of meningococcal disease in adolescence: prospective, matched-cohort study. Pediatrics..

[29] Stein-Zamir C, Shoob H, Sokolov I, Kunbar A, Abramson N, Zimmerman D (2014). The clinical features and long-term sequelae of invasive meningococcal disease in children. Pediatr Infect Dis J.

[30] Svendsen MB, Ring Kofoed I, Nielsen H, Schønheyder HC, Bodilsen J (2020). Neurological sequelae remain frequent after bacterial meningitis in children. Int J Pediatr.

[31] Sadarangani M, Scheifele DW, Halperin SA, Vaudry W, Le SN, Tsang R (2015). Outcomes of invasive meningococcal disease in adults and children in Canada between 2002 and 2011: a prospective cohort study. Clin Infect Dis.

[32] Fairfax A, Brehaut J, Colman I, Sikora L, Kazakova A, Chakraborty P (2019). A systematic review of the association between coping strategies and quality of life among caregivers of children with chronic illness and/or disability. BMC Pediatr.

[33] Christensen H, Trotter CL, Hickman M, Edmunds WJ (2014). Re-evaluating cost effectiveness of universal meningitis vaccination (Bexsero) in England: modelling study. BMJ.

[34] Duevel JA, Hasemann L, Peña-Longobardo LM, Rodríguez-Sánchez B, Aranda-Reneo I, Oliva-Moreno J (2020). Considering the societal perspective in economic evaluations: a systematic review in the case of depression. Heal Econ Rev.

[35] Darton T, Guiver M, Naylor S, Jack DL, Kaczmarski EB, Borrow R (2009). Severity of meningococcal disease associated with genomic bacterial load. Clin Infect Dis.

[36] Karve S, Misurski D, Miller J, Davis KL (2011). Costs of sequelae associated with invasive meningococcal disease: findings from a US managed care population. Health Outcomes Res Med.

[37] Davis KL, Misurski D, Miller J, Karve S (2011). Cost impact of complications in meningococcal disease: evidence from a United States managed care population. Hum Vaccin.

[38] Czeisler MÉ, Rohan EA, Melillo S (2021). Mental health among parents of children aged <18 years and unpaid caregivers of adults during the COVID-19 pandemic — United States, December 2020 and February–⁠march 2021. MMWR Morb Mortal Wkly Rep.

[39] Gallacher SD, Seaton A. Meningococcal meningitis and COVID-19 co-infection. BMJ Case Rep. 2020;13(8). 10.1136/bcr-2020-237366. 10.1136/bcr-2020-237366 PMC7449365 32843469

[40] Amin-Chowdhury Z, Aiano F, Mensah A, Sheppard CL, Litt D, Fry NK (2021). Impact of the coronavirus disease 2019 (COVID-19) pandemic on invasive pneumococcal disease and risk of pneumococcal coinfection with severe acute respiratory syndrome coronavirus 2 (SARS-CoV-2): prospective National Cohort Study. England Clin Infect Dis.

[41] Alderson MR, Arkwright PD, Bai X, Black S, Borrow R, Caugant DA (2022). Surveillance and control of meningococcal disease in the COVID-19 era: a global meningococcal initiative review. J Inf Secur.

[42] Brueggemann AB, Jansen van Rensburg MJ, Shaw D, McCarthy ND, Jolley KA, Maiden MCJ (2021). Changes in the incidence of invasive disease due to Streptococcus pneumoniae, Haemophilus influenzae, and Neisseria meningitidis during the COVID-19 pandemic in 26 countries and territories in the invasive respiratory infection surveillance initiative: a prospective analysis of surveillance data. Lancet Digit Health.

[43] Clark S, Campbell H, Mensah AA, Lekshmi A, Walker A, Ribeiro S et al. An Increase in Group B Invasive Meningococcal Disease Among Adolescents and Young Adults in England Following Easing of COVID-19 Containment Measures. 2021. 10.2139/ssrn.3998164. Accessed 10-3-2022.

