# Supplementary material for: Range of invasive meningococcal disease sequelae and health economic application – a systematic and clinical review
Source: BMC Public Health. 2022 May 31;22:1078. doi: 10.1186/s12889-022-13342-2 (PMC9153861; doi:10.1186/s12889-022-13342-2)
Supplement: Supplementary file 1 — Additional file 1: Supplementary File 1. Systematic literature review. Fig. S1.1. Plain Language Summary. Table S1.1. Search strategies (A. Observational / B. Health economic). Table S1.2. PICOS inclusion/exclusion selection criteria. Fig. S1.2. PRISMA flow diagram – observational study searches. Fig. S1.3. PRISMA flow diagram – health economic study searches. Table S1.3. Completed 2009 PRISMA checklist. [file 12889_2022_13342_MOESM1_ESM.docx]

**Supplementary file 1: Systematic literature review**

**Figure S1.1** Plain Language summary

**Table S1.1.** Search strategies (A. Observational / B. Economic evaluation)

**Table S1.2.** PICOS inclusion/exclusion selection criteria

**Figure S1.2** PRISMA flow diagram – observational study searches

**Figure S1.3** PRISMA flow diagram – health economic study searches

**Table S1.3.** Completed 2009 PRISMA checklist

**References** (for the 66 observational studies and 34 economic evaluations)

**Figure 1.1 Plain Language Summary**

**
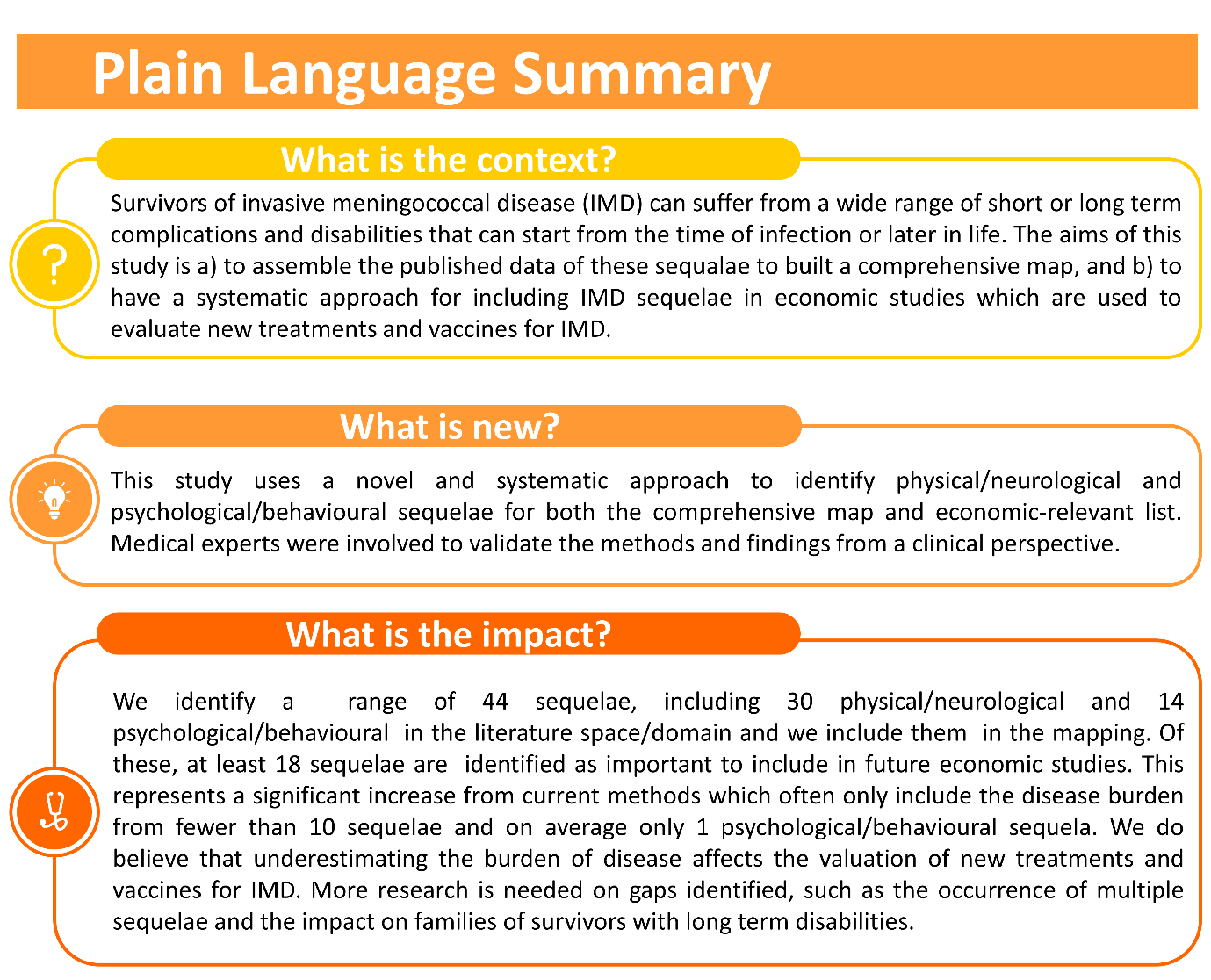
**

**Table S1.1. Search strategies**

1. Observational studies – search dates: from 1 August 2016 – 2 April 2020 (filters: Humans, Dutch, English, German, Spanish, French), systematic literature review (SLR) update of a previous SLR from 2001-2016

| # | Medline (PubMed) | Embase |
| --- | --- | --- |
| #1 | Neisseria meningitidis [mh] | Neisseria meningitidis'/exp |
| #2 | Meningococcal infections [mh] | ‘Meningococcosis'/exp |
| #3 | Neisseria meningitidis | ‘Neisseria meningitides' |
| #4 | Meningococcal disease | Meningococcal disease' |
| #5 | Meningococcal infection | 'Meningococcal infection' |
| #6 | ((((#1) OR #2) OR #3) OR #4) OR #5 | #1 OR #2 OR #3 OR #4 OR #5 |
| #7 | Quality of life [mh:noexp] | Quality of life'/exp |
| #8 | Quality-adjusted life years [mh:noexp] | Quality adjusted life year'/exp |
| #9 | Health Status [mh] | 'Health status'/exp |
| #10 | Patient preference [mh] | Patient preference'/exp |
| #11 | (Utility or utilities) | Utility OR utilities |
| #12 | Preference* | Preference* |
| #13 | Burden of illness [mh:noexp] | 'Burden of illness' |
| #14 | (Disease AND burden) | Disease NEAR/3 burden |
| #15 | Health state | 'Health state' |
| #16 | Sequela* | Sequela* |
| #17 | Complication* | Complication* |
| #18 | Mortality [mh] | Mortality'/exp |
| #19 | (((((((((((#7) OR #8) OR #9) OR #10) OR #11) OR #12) OR #13) OR #14) OR #15) OR #16) OR #17) OR #18 | #7 - #18/OR |
| #20 | ((#6) AND #19) | #6 AND #19 |

1. Economic evaluation - search dates: from 10 October 2016 – 2 June 2020 (filters: Humans, English, French), SLR update of a previous comprehensive search from 2001-2016

| # | Medline (PubMed) | Embase |  |
| --- | --- | --- | --- |
| Disease and intervention | |  |  |
| #1 | “Meningococcal Infections”[MeSH] | ‘Bacterial meningitis’/exp | MeSH descriptor: [Meningococcal Infections] explode all trees |
| #2 | Meningococcal*[TW] | ‘Meningococcal*’:ab,ti | MeSH descriptor: [Meningitis] explode all trees |
| #3 | Meningitis[MeSH] | ‘Meningitis’/exp | Meningococcal*:ti,ab,kw |
| #4 | Meningitis*[TW] | ‘Meningitis*’:ab,ti | Meningitis*:ti,ab,kw |
| #5 | Immunization[MeSH] | ‘Immunization’/exp | MeSH descriptor: [Immunization] explode all trees |
| #6 | Immuni*[TW] | ‘Immuni*’:ab,ti | Immuni*:ti,ab,kw |
| #7 | “Immunization programs”[MeSH] | ‘Preventive health services’/exp | MeSH descriptor: [Preventive Health Services] explode all trees |
| #8 | “Preventive health services” [MeSH] | ‘Vaccine’/exp | MeSH descriptor: [Vaccines] explode all trees |
| #9 | Vaccines[MeSH] | ‘Vaccination’/exp | MeSH descriptor: [Vaccination] explode all trees |
| #10 | Vaccination[MeSH] | ‘Vaccin*’:ab,ti | Vaccin*:ti,ab,kw |
| #11 | Vaccin*[tw] | ‘Inoculat*’:ab,ti | Inoculat*:ti,ab,kw |
| #12 | Inoculat*[tw] | #1 OR #2 OR #3 OR #4 | #1 or #2 or #3 or #4 |
| #13 | #1 OR #2 OR #3 OR #4 | #5 OR #6 OR #7 OR #8 OR #9 OR #10 OR #11 | #5 or #6 or #7 or #8 or #9 or #10 or #11 |
| #14 | #5 OR #6 OR #7 OR # 8 OR #9 OR #10 OR #11 OR #12 | #12 AND #13 | #12 and #13 |
| #15 | #13 AND #14 |  |  |
| Outcomes/study type | |  |  |
| #16 | Economics[MeSH:noexp] | 'Socioeconomics'/exp OR 'socioeconomics':ab,ti OR 'socioeconomics'/de | Filter: 2001 AND “economic evaluation” * |
| #17 | "Costs and cost analysis"[MeSH:noexp] | 'Cost benefit analysis'/exp OR 'cost benefit analysis':ab,ti OR 'cost benefit analysis'/de |  |
| #18 | “Cost allocation”[MeSH:noexp] | 'Cost effectiveness analysis'/exp OR 'cost effectiveness analysis':ab,ti OR 'cost effectiveness analysis'/de |  |
| #19 | “Cost-benefit analysis”[MeSH:noexp] | 'Cost of illness'/exp OR 'cost of illness':ab,ti OR 'cost of illness'/de |  |
| #20 | “Cost control”[MeSH:noexp] | 'Cost control'/exp OR 'cost control':ab,ti OR 'cost control'/de |  |
| #21 | “Cost savings”[MeSH:noexp] | 'Economic aspect'/exp OR 'economic aspect':ab,ti OR 'economic aspect'/de |  |
| #22 | “Cost of illness”[MeSH:noexp] | 'Financial management'/exp OR 'financial management':ab,ti OR 'financial management'/de |  |
| #23 | "Cost sharing"[MeSH:noexp] | 'Drug cost'/exp OR 'drug cost':ab,ti OR 'drug cost'/de |  |
| #24 | "Deductibles and coinsurance"[MeSH:noexp] | 'Health care cost'/exp OR 'health care cost':ab,ti OR 'health care cost'/de |  |
| #25 | “Medical savings accounts”[MeSH:noexp] | 'Health care financing'/exp OR 'health care financing':ab,ti OR 'health care financing'/de |  |
| #26 | “Health care costs”[MeSH:noexp] | 'Health economics'/exp OR 'health economics':ab,ti OR 'health economics'/de |  |
| #27 | “Direct service costs”[MeSH:noexp] | 'Hospital cost'/exp OR 'hospital cost':ab,ti OR 'hospital cost'/de |  |
| #28 | “Drug costs”[MeSH:noexp] | 'Cost minimization analysis'/exp OR 'cost minimization analysis':ab,ti OR 'cost minimization analysis'/de |  |
| #29 | “Employer health costs”[MeSH:noexp] | 'Fiscal'/exp OR 'fiscal'/de OR 'fiscal':ab,ti |  |
| #30 | “Hospital costs”[MeSH:noexp] | 'Financial':ab,ti OR 'financial'/de OR 'financial'/exp |  |
| #31 | “Health expenditures”[MeSH:noexp] | 'Fiscal' OR 'financial' OR 'financial':ab,ti OR funding:ab,ti OR 'funding'/exp OR 'funding'/de OR 'finance'/exp OR 'finance':ab,ti OR 'finance'/de |  |
| #32 | “Capital expenditures”[MeSH:noexp] | (Cost AND estimate*):ab,ti |  |
| #33 | “Value of life”[MeSH:noexp] | Cost:ab,ti AND variable*:ab,ti |  |
| #34 | “Economics, hospital”[MeSH] | cost*:ab,ti AND unit:ab,ti |  |
| #35 | “Economics, medical”[MeSH] | #15 OR #16 OR #17 OR #18 OR #19 OR #20 OR #21 OR #22 OR #23 OR #24 OR #25 OR #26 OR #27 OR #28 OR #29 OR #30 OR #31 OR #32 OR #33 |  |
| #36 | “Economics, nursing”[MeSH:noexp] | #14 AND #34 |  |
| #37 | “Economics, pharmaceutical”[MeSH:noexp] | - |  |
| #38 | "Fees and charges"[MeSH] | - |  |
| #39 | Budgets[MESH] | - |  |
| #40 | Low[TW] AND cost*[TW] | - |  |
| #41 | High[TW] AND cost*[TW] | - |  |
| #42 | "Health?care"[tw] AND cost*[tw] | - |  |
| #43 | Fiscal[TIAB] OR funding[TIAB] OR financial[TIAB] OR finance[TIAB] | - |  |
| #44 | Cost[tw] AND estimate*[tw] | - |  |
| #45 | Cost[tw] AND variable*[tw] | - |  |
| #46 | Unit[tw] AND cost*[tw] | - |  |
| #47 | Economic*[TIAB] OR pharmacoeconomic*[TIAB] OR price*[TIAB] OR pricing*[TIAB] | - |  |
| #48 | #16 OR #17 OR #18 OR #19 OR 20 OR #21 OR #22 OR #23 OR #24 OR #25 OR #26 OR #27 OR #28 OR #29 OR #30 OR #31 OR #32 OR #33 OR #34 OR #35 OR #36 OR #37 OR #38 OR #39 OR #40 OR #41 OR #42 OR #43 OR #44 OR #45 OR #46 OR #47 | - |  |
| #49 | #48 AND #15 | - |  |
| Exclusion terms | |  |  |
| #50 | “Case reports”[PT] OR comment[PT] OR editorial[PT] OR historical article[PT] OR letter[PT] | 'Comment':it OR 'editorial':it OR 'letter':it |  |
| #51 | Animals[MeSH] NOT humans[MeSH] | 'Animals'/exp NOT 'humans'/exp |  |
| #52 | #50 OR #51 | #36 OR #51 |  |
| #53 | #49 NOT #52 | #35 NOT #52 |  |

**Table S1.2. PICOS inclusion/exclusion selection criteria**

| Criteria | OS | | HE | |
| --- | --- | --- | --- | --- |
|  | **Inclusion** | **Exclusion** | **Inclusion** | **Exclusion** |
| Patients | IMD survivors all ages in HIC with clinical presentation of meningitis, septicaemia, septic shock or meningococcaemia, regardless of pathogen if a significant proportion was attributable to *N. meningitidis.* |  | All ages eligible for IMD vaccination regardless of comorbidities | No further restrictions were applied |
| Intervention | Not of specific interest |  | All IMD vaccination strategies (including no vaccination). | All pharmacological and non-pharmacological intervention focused on treating or curing the disease |
| Comparator | Not of specific interest |  | All IMD vaccination strategies (including no vaccination) | - |
| Outcomes* | IMD sequelae and health related quality of life in patients and caregivers |  | Incremental costs and QALYs; any other measure of effectiveness reported together with costs | Studies with cost only or epidemiology only outcomes |
| Study design | Primary studies including prospective cohort, case-controlled studies, retrospective chart reviews. | SLRs, case reports, medication or vaccination studies and epidemiologic or economic studies | Full economic evaluations with incremental costs or QALYs (or other measures of effectiveness with costs).  CBA studies with clinical outcomes (only if study was quantified in monetary units).  CEA considered if based on both modelling techniques or RCT | Burden of illness, costing, utility, epidemiological and clinical/biological studies.  Epidemiology models without cost component, studies of disease transmission without an economic component, studies of diagnostic samples and diagnostic performance |

**CBA** cost-benefit analysis; **CEA** cost-effectiveness analysis; **HE** health economics; **HIC** high income countries; **IMD** invasive meningococcal disease; **OS** observational studies; **PICOS** patients, intervention, comparator, outcomes, study design; **QALY**: quality-adjusted life-year; **RCT**: randomised controlled trial; **SLR** systematic literature review

**OECD high-income countries include:** Australia, Austria, Belgium, Canada, Chile, Czech Republic, Denmark, Estonia, Finland, France, Germany, Greece, Iceland, Ireland, Israel, Italy, Japan, Luxembourg, Netherlands, New Zealand, Norway, Poland, Portugal, Slovakia, Slovenia, South Korea, Spain, Sweden, Switzerland, United Kingdom, United States

*All outcomes relevant to understanding the disease burden of the patient and beyond was considered, however the outcomes considered in this study focused on IMD sequelae

**Figure S1.1 PRISMA flow diagram – observational study search 2001-2020**

Records identified through database searches N=3862

Duplicates removed N=216

Titles/Abstracts screened N=3646

Records excluded N=3579

Full text articles assessed for eligibility N=67

Full text excluded N=1

Records after duplicates removed N=3646

Studies included N=66

**Figure S1.2 PRISMA flow diagram – economic evaluation search 2001-2020**

Records identified through database searches N=4994

Duplicates removed N=708

Titles/Abstracts screened N=4286

Records excluded N=4193

Full text articles assessed for eligibility N=93

Full text articles N=59

Records after duplicates removed N=4286

Studies included for data extraction N=34

**Table S1.3. Completed 2009 PRISMA Checklist**

| **Section/topic** | **#** | **Checklist item** | **Reported on page #** |
| --- | --- | --- | --- |
| **TITLE** | | |  |
| Title | 1 | Identify the report as a systematic review, meta-analysis, or both. | L2-3 |
| **ABSTRACT** | | |  |
| Structured summary | 2 | Provide a structured summary including, as applicable: background; objectives; data sources; study eligibility criteria, participants, and interventions; study appraisal and synthesis methods; results; limitations; conclusions and implications of key findings; systematic review registration number. | L19-38 |
| **INTRODUCTION** | | |  |
| Rationale | 3 | Describe the rationale for the review in the context of what is already known. | L72-80 |
| Objectives | 4 | Provide an explicit statement of questions being addressed with reference to participants, interventions, comparisons, outcomes, and study design (PICOS). | L81-84 |
| **METHODS** | | |  |
| Protocol and registration | 5 | Indicate if a review protocol exists, if and where it can be accessed (e.g., Web address), and, if available, provide registration information including registration number. | NA |
| Eligibility criteria | 6 | Specify study characteristics (e.g., PICOS, length of follow-up) and report characteristics (e.g., years considered, language, publication status) used as criteria for eligibility, giving rationale. | Table S1.2 |
| Information sources | 7 | Describe all information sources (e.g., databases with dates of coverage, contact with study authors to identify additional studies) in the search and date last searched. | L88-94 |
| Search | 8 | Present full electronic search strategy for at least one database, including any limits used, such that it could be repeated. | Table S1.1A/B |
| Study selection | 9 | State the process for selecting studies (i.e., screening, eligibility, included in systematic review, and, if applicable, included in the meta-analysis). | L96-103 |
| Data collection process | 10 | Describe method of data extraction from reports (e.g., piloted forms, independently, in duplicate) and any processes for obtaining and confirming data from investigators. | L103-105 |
| Data items | 11 | List and define all variables for which data were sought (e.g., PICOS, funding sources) and any assumptions and simplifications made. | L104-105  Table S1.2 |
| Risk of bias in individual studies | 12 | Describe methods used for assessing risk of bias of individual studies (including specification of whether this was done at the study or outcome level), and how this information is to be used in any data synthesis. | L100, L103  Fig 1 |
| Summary measures | 13 | State the principal summary measures (e.g., risk ratio, difference in means). | L110-115  L126-129 |
| Synthesis of results | 14 | Describe the methods of handling data and combining results of studies, if done, including measures of consistency (e.g., I^2^) for each meta-analysis. | NA |
| Risk of bias across studies | 15 | Specify any assessment of risk of bias that may affect the cumulative evidence (e.g., publication bias, selective reporting within studies). | L132-134 |
| Additional analyses | 16 | Describe methods of additional analyses (e.g., sensitivity or subgroup analyses, meta-regression), if done, indicating which were pre-specified. | NA |
| **RESULTS** | | |  |
| Study selection | 17 | Give numbers of studies screened, assessed for eligibility, and included in the review, with reasons for exclusions at each stage, ideally with a flow diagram. | Fig S1.1, Fig S1.2 |
| Study characteristics | 18 | For each study, present characteristics for which data were extracted (e.g., study size, PICOS, follow-up period) and provide the citations. | Figs 2-4  Table 1  Table S2.2 |
| Risk of bias within studies | 19 | Present data on risk of bias of each study and, if available, any outcome level assessment (see item 12). | Table 1 |
| Results of individual studies | 20 | For all outcomes considered (benefits or harms), present, for each study: (a) simple summary data for each intervention group (b) effect estimates and confidence intervals, ideally with a forest plot. | Table 1  Table S2.2 |
| Synthesis of results | 21 | Present results of each meta-analysis done, including confidence intervals and measures of consistency. | NA |
| Risk of bias across studies | 22 | Present results of any assessment of risk of bias across studies (see Item 15). | Fig 1  Table 2.2 |
| Additional analysis | 23 | Give results of additional analyses, if done (e.g., sensitivity or subgroup analyses, meta-regression [see Item 16]). | NA |
| **DISCUSSION** | | |  |
| Summary of evidence | 24 | Summarize the main findings including the strength of evidence for each main outcome; consider their relevance to key groups (e.g., healthcare providers, users, and policy makers). | L232-237  L260-270 |
| Limitations | 25 | Discuss limitations at study and outcome level (e.g., risk of bias), and at review-level (e.g., incomplete retrieval of identified research, reporting bias). | L309-325 |
| Conclusions | 26 | Provide a general interpretation of the results in the context of other evidence, and implications for future research. | L335-342 |
| **FUNDING** | | |  |
| Funding | 27 | Describe sources of funding for the systematic review and other support (e.g., supply of data); role of funders for the systematic review. | L390-391 |

**References for the 66 observational studies** [1-66] and **34 economic evaluations** [67-100]

1. Erickson LJ, De Wals P, McMahon J, Heim S. Complications of meningococcal disease in college students. Clin Infect Dis. 2001;33(5):737-9. <https://doi.org/10.1086/322587>

2. Oostenbrink R, Maas M, Moons KGM, Moll HA. Sequelae after bacterial meningitis in childhood. Scand J Infect Dis. 2002;34(5):379-82. <https://doi.org/10.1080/00365540110080179>

3. Berg S, Trollfors B, Hugosson S, Fernell E, Svensson E. Long-term follow-up of children with bacterial meningitis with emphasis on behavioural characteristics. Eur J Pediatr. 2002;161(6):330-6. <https://doi.org/10.1007/s00431-002-0957-1>

4. van de Beek D, Schmand B, de Gans J, Weisfelt M, Vaessen H, Dankert J et al. Cognitive impairment in adults with good recovery after bacterial meningitis. J Infect Dis. 2002;186(7):1047-52. <https://doi.org/10.1086/344229>

5. Plötz FB, Uiterwaal CSPM, Riedijk M, van Vught AJ, van der Ent CK. Arteriële zuurstofsaturatie enkele jaren na een meningokokkensepsissyndroom: ernstige daling na maximale inspanning bij 6 van de 18 onderzochte kinderen. Ned Tijdschr Geneeskd. 2002 146(3):120-3.

6. Stovall SH, Schutze GE. Meningococcal infections in children from Arkansas. Pediatr Infect Dis J. 2002;21(5):366-70. <https://doi.org/10.1097/00006454-200205000-00003>

7. Goicoechea Saez M, Fullana Montoro AM, Momparler CP, Redondo Gallego MJ, Brines SJ, Bueno Canigral FJ. [Evolution of meningococcal infection among infant population in the autonomous community of Valencia (1996-2000). Effectiveness of A+C meningococcal vaccination]. Rev Esp Salud Publica. 2003;77(1):125-42.

8. Halket S, de Louvois J, Holt DE, Harvey D. Long term follow up after meningitis in infancy: behaviour of teenagers. Arch Dis Child. 2003;88(5):395-8. <https://doi.org/10.1136/adc.88.5.395>

9. Wellman MB, Sommer DD, McKenna J. Sensorineural hearing loss in postmeningitic children. Otol Neurotol. 2003;24(6):907-12. <https://doi.org/10.1097/00129492-200311000-00015>

10. Koomen I, Raat H, Jennekens-Schinkel A, Grobbee DE, Roord JJ, van Furth M. Academic and behavioral limitations and health-related quality of life in school-age survivors of bacterial meningitis. Qual Life Res. 2005;14(6):1563-72. <https://doi.org/10.1007/s11136-004-7706-z>

11. Dahlem P, de Jongh FHC, Griffioen RW, Bos AP, van Aalderen WMC. Respiratory sequelae after acute hypoxemic respiratory failure in children with meningococcal septic shock. Crit Care Shock. 2004;7(1):20-6.

12. Shears D, Nadel S, Gledhill J, Garralda ME. Short-term psychiatric adjustment of children and their parents following meningococcal disease. Pediatr Crit Care Med. 2005;6(1):39-43. <https://doi.org/10.1097/01.PCC.0000144705.81825.EE>

13. Slack R, Hawkins KC, Gilhooley L, Addison GM, Lewis MA, Webb NJ. Long-term outcome of meningococcal sepsis-associated acute renal failure. Pediatr Crit Care Med. 2005;6(4):477-9. <https://doi.org/10.1097/01.Pcc.0000163285.74452.23>

14. Kutz JW, Simon LM, Chennupati SK, Giannoni CM, Manolidis S. Clinical predictors for hearing loss in children with bacterial meningitis. Arch Otolaryngol Head Neck Surg. 2006;132(9):941-5. <https://doi.org/10.1001/archotol.132.9.941>

15. Bache CE, Torode IP. Orthopaedic sequelae of meningococcal septicemia. J Pediatr Orthop. 2006;26(1):135-9. <https://doi.org/10.1097/01.bpo.0000187991.71645.e7>

16. Van Dijk IAG, de Vries E. Meningococcal disease: Not always a fulminant course. Tijdschrift voor Kindergeneeskunde. 2006;74(3):97-100.

17. Hoogman M, van de Beek D, Weisfelt M, de Gans J, Schmand B. Cognitive outcome in adults after bacterial meningitis. J Neurol Neurosurg Psychiatry. 2007;78(10):1092-6. <https://doi.org/10.1136/jnnp.2006.110023>

18. Shears D, Nadel S, Gledhill J, Gordon F, Garralda ME. Psychiatric adjustment in the year after meningococcal disease in childhood. J Am Acad Child Adolesc Psychiatry. 2007;46(1):76-82. <https://doi.org/10.1097/01.chi.0000242234.83140.56>

19. Streharova A, Krcmery V, Kisac P, Kalavsky E, Holeckova K, Lesnakova A et al. Predictors of inferior outcome in community acquired bacterial meningitis. Neuro Endocrinol Lett. 2007;28(Suppl. 3):2-4.

20. Buysse CMP, Raat H, Hazelzet JA, Hop WC, Maliepaard M, Joosten KF. Surviving meningococcal septic shock: health consequences and quality of life in children and their parents up to 2 years after pediatric intensive care unit discharge. Crit Care Med. 2008;36(2):596-602. <https://doi.org/10.1097/01.CCM.0000299740.65484.CA>

21. Heckenberg SG, de Gans J, Brouwer MC, Weisfelt M, Piet JR, Spanjaard L et al. Clinical features, outcome, and meningococcal genotype in 258 adults with meningococcal meningitis: a prospective cohort study. Medicine (Baltimore). 2008;87(4):185-92. <https://doi.org/10.1097/MD.0b013e318180a6b4>

22. Vermunt LC, Buysse CM, Joosten KF, Hazelzet JA, Verhulst FC, Utens EM. Behavioural, emotional, and post-traumatic stress problems in children and adolescents, long term after septic shock caused by Neisseria meningitidis. Br J Clin Psychol. 2008;47(Part 3):251-63. <https://doi.org/10.1348/014466507x258868>

23. Douglas SA, Sanli H, Gibson WPR. Meningitis resulting in hearing loss and labyrinthitis ossificans - Does the causative organism matter? Cochlear Implants Int. 2008;9(2):90-6. <https://doi.org/10.1179/cim.2008.9.2.90>

24. Ritchi L, Jennekens-Schinkel A, Van Schooneveld M, Koomen I, Geenen R. Behaviour is not really at risk after surviving meningitis in childhood. Acta Paediatrica, International Journal of Paediatrics. 2008;97(4):438-41. <https://doi.org/10.1111/j.1651-2227.2008.00682.x>

25. Borg J, Christie D, Coen PG, Booy R, Viner RM. Outcomes of meningococcal disease in adolescence: prospective, matched-cohort study. Pediatrics. 2009;123(3):e502-e9. <https://doi.org/10.1542/peds.2008-0581>

26. Buysse CMP, Oranje AP, Zuidema E, Hazelzet JA, Hop WC, Diepstraten AF et al. Long-term skin scarring and orthopaedic sequelae in survivors of meningococcal septic shock. Arch Dis Child. 2009;94(5):381-6. <https://doi.org/10.1136/adc.2007.131862>

27. Cabellos C, Verdaguer R, Olmo M, Fernández-Sabé N, Cisnal M, Ariza J et al. Community-acquired bacterial meningitis in elderly patients: Experience over 30 years. Medicine. 2009;88(2):115-9. <https://doi.org/10.1097/MD.0b013e31819d50ef>

28. Dzupova O, Rozsypal H, Prochazka B, Benes J. Acute bacterial meningitis in adults: predictors of outcome. Scand J Infect Dis. 2009;41(5):348-54. <https://doi.org/10.1080/00365540902849391>

29. Schmand B, de Bruin E, de Gans J, van de Beek D. Cognitive functioning and quality of life nine years after bacterial meningitis. J Infect. 2010;61(4):330-4. <https://doi.org/10.1016/j.jinf.2010.07.006>

30. Vermunt LC, Buysse CM, Joosten KF, Hazelzet JA, Verhulst FC, Utens EM. Recovery in parents of children and adolescents who survived septic shock caused by Neisseria meningitidis: a cross-sectional study. Intensive Crit Care Nurs. 2010;26(3):128-37. <https://doi.org/10.1016/j.iccn.2010.01.001>

31. Vermunt LC, Buysse CM, Joosten KF, Duivenvoorden HJ, Hazelzet JA, Verhulst FC et al. Survivors of septic shock caused by Neisseria meningitidis in childhood: psychosocial outcomes in young adulthood. Pediatr Crit Care Med. 2011;12(6):e302-e9. <https://doi.org/10.1097/PCC.0b013e3182192d7f>

32. Gottfredsson M, Reynisson IK, Ingvarsson RF, Kristjansdottir H, Nardini MV, Sigurdsson JF et al. Comparative long-term adverse effects elicited by invasive group B and C meningococcal infections. Clin Infect Dis. 2011;53(9):e117-e24. <https://doi.org/10.1093/cid/cir500>

33. Sumpter R, Brunklaus A, McWilliam R, Dorris L. Health-related quality-of-life and behavioural outcome in survivors of childhood meningitis. Brain Inj. 2011;25(13-14):1288-95. <https://doi.org/10.3109/02699052.2011.613090>

34. Vartzelis G, Vasilopoulou V, Katsioulis A, Hadjichristodoulou C, Theodoridou M. Functional and behavioral outcome of bacterial meningitis in school-aged survivors. Pediatrics Int. 2011;53(3):300-2. <https://doi.org/10.1111/j.1442-200X.2011.03387.x>

35. Viner RM, Booy R, Johnson H, Edmunds WJ, Hudson L, Bedford H et al. Outcomes of invasive meningococcal serogroup B disease in children and adolescents (MOSAIC): a case-control study. Lancet Neurol. 2012;11(9):774-83. <https://doi.org/10.1016/S1474-4422(12)70180-1>

36. Herrero M, Alcalde M, Gómez B, Hernández JL, Sota M, Benito J et al. Invasive bacterial infections in a paediatric emergency department in the era of the heptavalent pneumococcal conjugate vaccine. Eur J Emerg Med. 2012;19(2):89-94. <https://doi.org/10.1097/MEJ.0b013e3283484bbc>

37. Bettinger JA, Scheifele DW, Le Saux N, Halperin SA, Vaudry W, Tsang R. The disease burden of invasive meningococcal serogroup B disease in Canada. Pediatr Infect Dis J. 2013;32(1):e20-5. <https://doi.org/10.1097/INF.0b013e3182706b89>

38. Moreno G, López D, Vergara N, Gallegos D, Advis MF, Loayza S. Clinical characterization of cases with meningococcal disease by W135 group in Chile, 2012. Revista Chilena de Infectologia. 2013;30(4):350-60. <https://doi.org/10.4067/s0716-10182013000400002>

39. Roed C, Omland LH, Skinhoj P, Rothman KJ, Sorensen HT, Obel N. Educational achievement and economic self-sufficiency in adults after childhood bacterial meningitis. JAMA. 2013;309(16):1714-21. <https://doi.org/10.1001/jama.2013.3792>

40. Stein-Zamir C, Shoob H, Sokolov I, Kunbar A, Abramson N, Zimmerman D. The clinical features and long-term sequelae of invasive meningococcal disease in children. Pediatr Infect Dis J. 2014;33(7):777-9. <https://doi.org/10.1097/INF.0000000000000282>

41. Wang B, Clarke M, Thomas N, Howell S, Afzali HH, Marshall H. The clinical burden and predictors of sequelae following invasive meningococcal disease in Australian children. Pediatr Infect Dis J. 2014;33(3):316-8. <https://doi.org/10.1097/INF.0000000000000043>

42. Kuchar E, Nitsch-Osuch A, Rorat M, Namani S, Pabianek D, Topczewska-Cabanek A et al. Etiology and complications of central nervous system infections in children treated in a pediatric intensive care unit in Poland. J Child Neurol. 2014;29(4):483-6. <https://doi.org/10.1177/0883073813477689>

43. Sadarangani M, Scheifele DW, Halperin SA, Vaudry W, Le SN, Tsang R et al. Outcomes of invasive meningococcal disease in adults and children in Canada between 2002 and 2011: a prospective cohort study. Clin Infect Dis. 2015;60(8):e27-e35. <https://doi.org/10.1093/cid/civ028>

44. Stoof SP, Rodenburg GD, Knol MJ, Rümke LW, Bovenkerk S, Berbers GA et al. Disease Burden of Invasive Meningococcal Disease in the Netherlands Between June 1999 and June 2011: A Subjective Role for Serogroup and Clonal Complex. Clin Infect Dis. 2015;61(8):1281-92. <https://doi.org/10.1093/cid/civ506>

45. Al-Janabi H, Van Exel J, Brouwer W, Trotter C, Glennie L, Hannigan L et al. Measuring Health Spillovers for Economic Evaluation: A Case Study in Meningitis. Health Econ. 2016;25(12):1529-44. <https://doi.org/10.1002/hec.3259>

46. Rivero-Calle I, Vilanova-Trillo L, Pardo-Seco J, Salvado LB, Quinteiro LI, Martinon-Torres F. The Burden of Pediatric Invasive Meningococcal Disease in Spain (2008-2013). Pediatr Infect Dis J. 2016;35(4):407-13. <https://doi.org/10.1097/INF.0000000000001048>

47. Ó Maoldomhnaigh C, Drew RJ, Gavin P, Cafferkey M, Butler KM. Invasive meningococcal disease in children in Ireland, 2001-2011. Arch Dis Child. 2016;101(12):1125-9. <https://doi.org/10.1136/archdischild-2015-310215>

48. van Veen KEB, Brouwer MC, van der Ende A, van de Beek D. Bacterial meningitis in hematopoietic stem cell transplant recipients: A population-based prospective study. Bone Marrow Transplantation. 2016;51(11):1490-5. <https://doi.org/10.1038/bmt.2016.181>

49. Kennedy ITR, van Hoek AJ, Ribeiro S, Christensen H, Edmunds WJ, Ramsay ME et al. Short-term changes in the health state of children with group B meningococcal disease: A prospective, national cohort study. PLoS One. 2017;12(5):e0177082. <https://doi.org/10.1371/journal.pone.0177082>

50. Pomar V, Benito N, López-Contreras J, Coll P, Gurguí M, Domingo P. Characteristics and outcome of spontaneous bacterial meningitis in patients with cancer compared to patients without cancer. Medicine (United States). 2017;96(19). <https://doi.org/10.1097/MD.0000000000006899>

51. Säll O, Stenmark B, Glimåker M, Jacobsson S, Mölling P, Olcén P et al. Clinical presentation of invasive disease caused by Neisseria meningitidis serogroup Y in Sweden, 1995 to 2012. Epidemiol Infect. 2017;145(10):2137-43. <https://doi.org/10.1017/s0950268817000929>

52. van Veen KE, Brouwer MC, van der Ende A, van de Beek D. Bacterial meningitis in alcoholic patients: A population-based prospective study. J Infect. 2017;74(4):352-7. <https://doi.org/10.1016/j.jinf.2017.01.001>

53. Lorton F, Chalumeau M, Assathiany R, Martinot A, Bucchia M, Roué JM et al. Vaccine-preventable severe morbidity and mortality caused by meningococcus and pneumococcus: A population-based study in France. Paediatr Perinat Epidemiol. 2018;32(5):442-7. <https://doi.org/10.1111/ppe.12500>

54. Pickering L, Jennum P, Ibsen R, Kjellberg J. Long-term health and socioeconomic consequences of childhood and adolescent onset of meningococcal meningitis. Eur J Pediatr. 2018;177(9):1309-15. <https://doi.org/10.1007/s00431-018-3192-0>

55. Boeddha NP, Schlapbach LJ, Driessen GJ, Herberg JA, Rivero-Calle I, Cebey-López M et al. Mortality and morbidity in community-acquired sepsis in European pediatric intensive care units: a prospective cohort study from the European Childhood Life-threatening Infectious Disease Study (EUCLIDS). Crit Care. 2018;22(1):143. <https://doi.org/10.1186/s13054-018-2052-7>

56. Contou D, Sonneville R, Canoui-Poitrine F, Colin G, Coudroy R, Pène F et al. Clinical spectrum and short-term outcome of adult patients with purpura fulminans: a French multicenter retrospective cohort study. Intensive Care Med. 2018;44(9):1502-11. <https://doi.org/10.1007/s00134-018-5341-3>

57. Gangoiti I, Valle JR, Sota M, Martinez-Indart L, Benito J, Mintegi S. Characteristics of children with microbiologically confirmed invasive bacterial infections in the emergency department. Eur J Emerg Med. 2018;25(4):274-80. <https://doi.org/10.1097/mej.0000000000000453>

58. Menichetti F, Fortunato S, Ricci A, Salani F, Ripoli A, Tascini C et al. Invasive Meningococcal Disease due to group C N. meningitidis ST11 (cc11): The Tuscany cluster 2015-2016. Vaccine. 2018;36(40):5962-6. <https://doi.org/10.1016/j.vaccine.2018.08.050>

59. Cabellos C, Pelegrín I, Benavent E, Gudiol F, Tubau F, Garcia-Somoza D et al. Invasive meningococcal disease: What we should know, before it comes back. Open Forum Infect Dis. 2019;6(3):ofz059. <https://doi.org/10.1093/ofid/ofz059>

60. Maturana Martínez D, Aguilera-Alonso D, García Mancebo J, Navarro ML, Hernández Sampelayo T, Rincón López EM. [Invasive meningococcal disease in children and adults in a tertiary level hospital. Recent epidemiology and prognostic factors]. An Pediatr (Barc). 2019;91(5):296-306. <https://doi.org/10.1016/j.anpedi.2018.12.005>

61. Elrod J, Mannhard D, Mohr C, Lienert C, Hagemann-Gysling K, Schiestl C et al. Plastic and Orthopaedic Interventions and Long-Term Sequelae in Children with Meningococcal Septicemia-40 Years of Experience at the University Children's Hospital Zurich. Eur J Pediatr Surg. 2019;29(5):462-9. <https://doi.org/10.1055/s-0038-1673705>

62. Labroca P, Chiesa G, Laroyenne I, Borrini L, Klotz R, Phan Sy Q et al. Quality of life assessment following amputation for septic shock: a long-term descriptive survey after symmetric peripheral gangrene. J Crit Care. 2019;53:231-5. <https://doi.org/10.1016/j.jcrc.2019.06.027>

63. Huang L, Heuer OD, Janßen S, Häckl D, Schmedt N. Clinical and economic burden of invasive meningococcal disease: Evidence from a large German claims database. PLoS ONE. 2020;15(1):e0228020. <https://doi.org/10.1371/journal.pone.0228020>

64. Tubiana S, Varon E, Biron C, Ploy MC, Mourvillier B, Taha MK et al. Community-acquired bacterial meningitis in adults: in-hospital prognosis, long-term disability and determinants of outcome in a multicentre prospective cohort. Clin Microbiol Infect. 2020;26(9):1192-200. <https://doi.org/10.1016/j.cmi.2019.12.020>

65. Svendsen MB, Ring Kofoed I, Nielsen H, Schønheyder HC, Bodilsen J. Neurological sequelae remain frequent after bacterial meningitis in children. Acta Paediatrica, International Journal of Paediatrics. 2020;109(2):361-7. <https://doi.org/10.1111/apa.14942>

66. Truong J, Levy C, Prot-Labarthe S, Nguyen HPK, Grimprel E, Faye A et al. Vaccine-preventable meningitis in French children with incorrect vaccination status from 2011 to 2013. Archives de Pediatrie. 2020;27(1):1-5. <https://doi.org/10.1016/j.arcped.2019.10.008>

67. Bos JM, Rümke HC, Welte R, Postma MJ, Jager JC. Health economics of a hexavalent meningococcal outer-membrane vesicle vaccine in children: potential impact of introduction in the Dutch vaccination program. Vaccine. 2001;20(1-2):202-7. <https://doi.org/10.1016/s0264-410x(01)00254-7>

68. Skull SA, Butler JR. Meningococcal vaccination for adolescents? An economic evaluation in Victoria. J Paediatr Child Health. 2001;37(s5):28-33. <https://doi.org/10.1046/j.1440-1754.2001.00652.x>

69. De Wals P, Erickson L. Economic analysis of the 1992-1993 mass immunization campaign against serogroup C meningococcal disease in Quebec. Vaccine. 2002;20(21-22):2840-4. <https://doi.org/10.1016/s0264-410x(02)00161-5>

70. Oostenbrink R, Oostenbrink JB, Moons KG, Derksen-Lubsen G, Essink-Bot ML, Grobbee DE et al. Cost-utility analysis of patient care in children with meningeal signs. Int J Technol Assess Health Care. 2002;18(3):485-96.

71. Scott RD, 2nd, Meltzer MI, Erickson LJ, De Wals P, Rosenstein NE. Vaccinating first-year college students living in dormitories for Meningococcal disease: an economic analysis. Am J Prev Med. 2002;23(2):98-105. <https://doi.org/10.1016/s0749-3797(02)00462-2>

72. Trotter CL, Edmunds WJ. Modelling cost effectiveness of meningococcal serogroup C conjugate vaccination campaign in England and Wales. BMJ. 2002;324(7341):809. <https://doi.org/10.1136/bmj.324.7341.809>

73. Ruedin HJ, Ess S, Zimmermann HP, Szucs T. Invasive meningococcal and pneumococcal disease in Switzerland: cost-utility analysis of different vaccine strategies. Vaccine. 2003;21(27-30):4145-52. <https://doi.org/10.1016/s0264-410x(03)00562-0>

74. Rancourt C, Grégoire JP, Simons W, Dostie A. Cost-benefit model comparing two alternative immunisation programmes against serogroup C meningococcal disease: for Quebec residents aged 2 months to 20 years. Pharmacoeconomics. 2003;21(6):429-42. <https://doi.org/10.2165/00019053-200321060-00006>

75. De Wals P, Deceuninck G, Boulianne N, De Serres G. Effectiveness of a mass immunization campaign using serogroup C meningococcal conjugate vaccine. JAMA. 2004;292(20):2491-4. <https://doi.org/10.1001/jama.292.20.2491>

76. Welte R, van den Dobbelsteen G, Bos JM, de Melker H, van Alphen L, Spanjaard L et al. Economic evaluation of meningococcal serogroup C conjugate vaccination programmes in The Netherlands and its impact on decision-making. Vaccine. 2004;23(4):470-9. <https://doi.org/10.1016/j.vaccine.2004.06.019>

77. Shepard CW, Ortega-Sanchez IR, Scott RD, 2nd, Rosenstein NE. Cost-effectiveness of conjugate meningococcal vaccination strategies in the United States. Pediatrics. 2005;115(5):1220-32. <https://doi.org/10.1542/peds.2004-2514>

78. Bos JM, Rümke HC, Welte R, Spanjaard L, van Alphen L, Postma MJ. Combination vaccine against invasive meningococcal B and pneumococcal infections: potential epidemiological and economic impact in the Netherlands. Pharmacoeconomics. 2006;24(2):141-53. <https://doi.org/10.2165/00019053-200624020-00004>

79. Trotter CL, Edmunds WJ. Reassessing the cost-effectiveness of meningococcal serogroup C conjugate (MCC) vaccines using a transmission dynamic model. Med Decis Making. 2006;26(1):38-47. <https://doi.org/10.1177/0272989x05284109>

80. De Wals P, Coudeville L, Trottier P, Chevat C, Erickson LJ, Nguyen VH. Vaccinating adolescents against meningococcal disease in Canada: a cost-effectiveness analysis. Vaccine. 2007;25(29):5433-40. <https://doi.org/10.1016/j.vaccine.2007.04.071>

81. Ortega-Sanchez IR, Meltzer MI, Shepard C, Zell E, Messonnier ML, Bilukha O et al. Economics of an adolescent meningococcal conjugate vaccination catch-up campaign in the United States. Clin Infect Dis. 2008;46(1):1-13. <https://doi.org/10.1086/524041>

82. Christensen H, Hickman M, Edmunds WJ, Trotter CL. Introducing vaccination against serogroup B meningococcal disease: an economic and mathematical modelling study of potential impact. Vaccine. 2013;31(23):2638-46. <https://doi.org/10.1016/j.vaccine.2013.03.034>

83. Hepkema H, Pouwels KB, van der Ende A, Westra TA, Postma MJ. Meningococcal serogroup A, C, W(1)(3)(5) and Y conjugated vaccine: a cost-effectiveness analysis in the Netherlands. PLoS One. 2013;8(5):e65036. <https://doi.org/10.1371/journal.pone.0065036>

84. Pouwels KB, Hak E, van der Ende A, Christensen H, van den Dobbelsteen GP, Postma MJ. Cost-effectiveness of vaccination against meningococcal B among Dutch infants: Crucial impact of changes in incidence. Hum Vaccin Immunother. 2013;9(5):1129-38. <https://doi.org/10.4161/hv.23888>

85. Christensen H, Trotter CL, Hickman M, Edmunds WJ. Re-evaluating cost effectiveness of universal meningitis vaccination (Bexsero) in England: modelling study. BMJ. 2014;349:g5725. <https://doi.org/10.1136/bmj.g5725>

86. Hanquet G, Christensen H, Agnew E, Trotter C, Robays J, Dubois C et al. A quadrivalent vaccine against serogroup B meningococcal disease: a cost-effectiveness study. Belgian Health Technology Assessment (HTA) KCE report 2312014.

87. Tu HA, Deeks SL, Morris SK, Strifler L, Crowcroft N, Jamieson FB et al. Economic evaluation of meningococcal serogroup B childhood vaccination in Ontario, Canada. Vaccine. 2014;32(42):5436-46. <https://doi.org/10.1016/j.vaccine.2014.07.096>

88. Tirani M, Meregaglia M, Melegaro A. Health and economic outcomes of introducing the new MenB vaccine (Bexsero) into the Italian routine infant immunisation programme. PLoS One. 2015;10(4):e0123383. <https://doi.org/10.1371/journal.pone.0123383>

89. Simon MS, Weiss D, Geevarughese A, Kratz MM, Cutler B, Gulick RM et al. Cost-Effectiveness of Meningococcal Vaccination Among Men Who Have Sex With Men in New York City. J Acquir Immune Defic Syndr. 2016;71(2):146-54. <https://doi.org/10.1097/qai.0000000000000822>

90. Christensen H, Irving T, Koch J, Trotter CL, Ultsch B, Weidemann F et al. Epidemiological impact and cost-effectiveness of universal vaccination with Bexsero((R)) to reduce meningococcal group B disease in Germany. Vaccine. 2016;34(29):3412-9. <https://doi.org/10.1016/j.vaccine.2016.04.004>

91. Gasparini R, Landa P, Amicizia D, Icardi G, Ricciardi W, de Waure C et al. Vaccinating Italian infants with a new multicomponent vaccine (Bexsero(R)) against meningococcal B disease: A cost-effectiveness analysis. Hum Vaccin Immunother. 2016;12(8):2148-61. <https://doi.org/10.1080/21645515.2016.1160177>

92. Ginsberg GM, Block C, Stein-Zamir C. Cost-utility analysis of a nationwide vaccination programme against serogroup B meningococcal disease in Israel. Int J Public Health. 2016;61(6):683-92. <https://doi.org/10.1007/s00038-016-0821-0>

93. Lecocq H, Parent du Chatelet I, Taha MK, Levy-Bruhl D, Dervaux B. Epidemiological impact and cost-effectiveness of introducing vaccination against serogroup B meningococcal disease in France. Vaccine. 2016;34(19):2240-50. <https://doi.org/10.1016/j.vaccine.2016.03.020>

94. Christensen H, Trotter CL. Modelling the cost-effectiveness of catch-up 'MenB' (Bexsero) vaccination in England. Vaccine. 2017;35(2):208-11. <https://doi.org/10.1016/j.vaccine.2016.11.076>

95. De Wals P, Zhou Z. Cost-effectiveness Comparison of Monovalent C Versus Quadrivalent ACWY Meningococcal Conjugate Vaccination in Canada. Pediatr Infect Dis J. 2017;36(7):e203-e7. <https://doi.org/10.1097/inf.0000000000001512>

96. Delea TE, Weycker D, Atwood M, Neame D, Alvarez FP, Forget E et al. Cost-effectiveness of alternate strategies for childhood immunization against meningococcal disease with monovalent and quadrivalent conjugate vaccines in Canada. PLoS One. 2017;12(5):e0175721. <https://doi.org/10.1371/journal.pone.0175721>

97. Leeds IL, Namasivayam V, Bamogo A, Sankhla P, Thayer WM. Cost Effectiveness of Meningococcal Serogroup B Vaccination in College-Aged Young Adults. Am J Prev Med. 2019;56(2):196-204. <https://doi.org/10.1016/j.amepre.2018.09.020>

98. Sevilla J, Tortorice D, Kantor D, Bloom DE, Hogea C, Beck E. PIN43 Lifecycle-model-based economic evaluation of infant meningitis B vaccination in the UK. ISPOR; 2019; Copenhagen, Denmark Value in Health. 2019;22:S647.

99. Si S, Zomer E, Fletcher S, Lee J, Liew D. Cost-effectiveness of meningococcal polysaccharide serogroups A, C, W-135 and Y conjugate vaccine in Australian adolescents. Vaccine. 2019;37(35):5009-15. <https://doi.org/10.1016/j.vaccine.2019.07.008>

100. Breton M-C, Huang L, Snedecor SJ, Cornelio N, Fanton-Aita F. Cost-effectiveness of alternative strategies for vaccination of adolescents against serogroup B IMD with the MenB-FHbp vaccine in Canada. Can J Public Health. 2020;111(2):182-92. <https://doi.org/10.17269/s41997-019-00275-4>
