# Supplementary material for: Range of invasive meningococcal disease sequelae and health economic application – a systematic and clinical review
Source: BMC Public Health. 2022 May 31;22:1078. doi: 10.1186/s12889-022-13342-2 (PMC9153861; doi:10.1186/s12889-022-13342-2)
Supplement: Supplementary file 2 — Additional file 2: Supplementary File 2. Data extraction and expert validation. Table S2.1. Pre-defined list of IMD sequelae with definitions, to which reported sequelae are mapped – expert validation comments. Table S2.2. Sequelae data extraction from high-rated studies (highest SIGN rating and highest number of sequelae reported). Table S2.3. IMD sequelae for comprehensive map: selected percentage (range across studies) – expert validation comments. Table S2.4. IMD sequelae relevant for economic evaluation: selected percentage (range across studies) – expert validation comments. [file 12889_2022_13342_MOESM2_ESM.docx]

**Supplementary file 2: Data extraction and expert validation**

**Table S2.1.** Pre-defined list of IMD sequelae with definitions, to which reported sequelae are mapped – expert validation comments

**Table S2.2.** Sequelae data extraction from high-rated studies (highest SIGN rating and highest number of sequelae reported)

**Table S2.3.** IMD sequelae for comprehensive map: selected percentage (range across studies) – expert validation comments

**Table S2.4.** IMD sequelae relevant for economic evaluation: selected percentage (range across studies) – expert validation comments

**References**

**Table S2.1. Pre-defined list of IMD sequelae with definitions, to which reported sequelae are mapped – expert validation comments**

The pre-defined overall list of all possible IMD sequelae used for mapping literature outcomes, for consistency and comparability, with assumptions for each sequela definition provided.

Expert validation: **1**= Agree with assumptions and well defined; **2**= Assumptions clinically representative; **3**= Any sequelae missing or misrepresented clinically

| Category | Assumptions within the definitions | Expert validation 1 | | | Expert validation 2 | | | Expert validation 3 | | |  |
| --- | --- | --- | --- | --- | --- | --- | --- | --- | --- | --- | --- |
| **Physical** |  | **1** | **2** | **3** | **1** | **2** | **3** | **1** | **2** | **3** | |
| *Dermatological condition* | |  |  |  |  |  |  |  |  |  | |
| Skin scarring (with/without grafting) |  | ✓ | ✓ | Colours change due to the scars (dark), invasive scars such as keloids | ✓ |  |  | Consider pain or cosmetic procedures |  |  | |
| Other and non-specified skin disease (e.g., skin necrosis, eczema, psoriasis) | Includes: skin lesions, surgical debridement | ✓ | ✓ |  | ✓ | I would include here skin pigmentation changes, but not psoriasis | Skin ulceration and decubitus related to prothesis | Consider pain or cosmetic procedures |  |  | |
| *Musculoskeletal deficiency (bone, muscle, joint)* | | | |  |  |  |  |  |  |  | |
| Arthritis |  | ✓ | ✓ |  | ✓ |  | Arthralgia is also possible, particularly with growth | ✓ |  |  | |
| Limb deficiency/deformities |  | ✓ | ✓ |  | ✓ |  |  | ✓ |  |  | |
| Amputation |  | ✓ | ✓ |  | ✓ |  |  | Define and extend? |  |  | |
| Other and non-specified musculoskeletal deficiencies | Includes: osteomyelitis and opisthotonos (as it refers to deformities in other parts of the body not just the limbs) | ✓ | ✓ |  | ✓ |  |  | ✓ |  |  | |
| *Renal condition* |  | *✓* | *✓* |  | ✓ | *Maybe within the subheading, but you may consider renal scarring, renal insufficiency. Important also, adrenal insufficiency (Waterhouse-Friderichsen)* | | *Consider chronic renal failure?* |  |  | |
| *Haematological condition* | | | |  |  |  |  | ✓ |  |  | |
| Anaemia |  | ✓ | ✓ |  | ✓ |  |  | ✓ |  |  | |
| Other and non-specified haematological conditions | Includes: conditions such as stroke/other vascular conditions included | ✓ | ✓ |  | ✓ |  |  | ✓ |  |  | |
| *Vascular condition* | | | |  |  |  |  | ✓ |  |  | |
| Raynaud phenomenon symptoms |  | ✓ | ✓ |  | ✓ |  |  | ✓ |  |  | |
| Venous thrombosis |  | ✓ | ✓ |  | ✓ |  |  | ✓ |  |  | |
| Vasculitis |  | ✓ | ✓ |  | ✓ |  |  | ✓ |  |  | |
| Other and non-specified vascular conditions | Includes: impaired liver functions | ✓ | ✓ |  | ✓ |  |  | ✓ |  |  | |
| Cranial nerve palsy | Is captured within neurological and under cerebral palsies category | ✓ | ✓ |  | ✓ |  |  | ✓ |  |  | |
| *Pulmonary condition* | *Includes: pulmonary hypertension, ARDS/respiratory sequelae/lung functioning* | *✓* | *✓* |  | ✓ |  |  | ✓ |  |  | |
| Other and non-specific physical conditions |  | ✓ | ✓ |  | ✓ |  |  | ✓ |  |  | |
| **Neurological** |  |  |  |  |  |  |  |  |  |  | |
| Sensory system deficits | | |  |  |  |  |  | ✓ |  |  | |
| Blindness/severe visual impairment |  | ✓ | ✓ | Visual impairment more frequent than blindness - although both are not that frequent | ✓ |  |  | ✓ |  |  | |
| Other visual impairments |  |  |  |  | ✓ |  |  | ✓ |  |  | |
| Hearing loss – severe/profound bilateral (cochlear implant) |  | ✓ | ✓ | Less frequent / depend on the timing of treatment and how the patient did respond | ✓ |  |  | ✓ |  |  | |
| Hearing loss – moderate bilateral |  | ✓ | ✓ | Depends on if the patient is treated early enough | ✓ |  |  | ✓ |  |  | |
| Hearing loss – unilateral/hearing impairment | If non-specific, hearing loss is placed under this category | ✓ | ✓  Can happen |  | ✓ |  |  | ✓ |  |  | |
| Tinnitus |  |  |  |  | ✓ |  |  | ✓ |  |  | |
| Numbness |  | ✓ | ✓  Can be transient/ permanent |  | ✓ |  |  | ✓ |  |  | |
| Paraesthesia/reduced sensitivity |  | ✓ | ✓ |  | ✓ |  |  | ✓ |  |  | |
| Other and non-specified sensory system deficits |  |  |  |  | ✓ |  |  | ✓ |  |  | |
| *Motor deficits* |  |  |  |  |  |  |  |  |  |  | |
| Palsy | This is the broader category to capture paralysis, cerebral palsies etc | NA | NA |  | ✓ |  |  | ✓ |  |  | |
| Muscle weakness | Includes: hemiparesis, focal neurological deficits (defined as hemiparesis; cranial nerve deficits,  increased or decreased reflexes of arms or legs, increased or decreased tonus of arms or legs, and ataxia; sensory loss in 1 arm, paresis of 1 arm, paraesthesia of the foot and/ or hand, and resting and intention tremor of both hands), a mild transitory left-sided weakness | ✓ | ✓ |  | ✓ |  |  | ✓ |  |  | |
| Movement coordination/balance deficits | Includes: vestibular syndrome e.g., vertigo (as it related to the inner ear system) | ✓ | ✓  Appropriate follow-up is needed for final diagnosis |  | ✓ |  |  | ✓ |  |  | |
| Spasticity |  |  |  |  | ✓ |  |  | ✓ |  |  | |
| Other and non-specific motor deficits |  |  |  |  | ✓ |  |  | ✓ |  |  | |
| *Communication disorders (speech, language, communication)* | *Includes: aphasia* | *✓* | *✓* |  | ✓ |  |  | ✓ |  |  | |
| *Intellectual disability* |  |  |  |  | *Beyond objective physical or neurological sequelae, IMD may have impact on health-related quality of life which is measured in several studies and should be captured in some manner, as it, or within the psychological?* | | | ✓ |  |  | |
| Mental retardation/low IQ |  | ✓ | ✓ |  | ✓ |  |  | ✓ |  |  | |
| Mild IQ loss (70-85) |  | ✓ | Dependent on what age and whether we are speaking about regression? Or the lack of further development vs, appropriate development vs age |  | ✓ |  |  | ✓ |  |  | |
| Learning disabilities | Includes: cognitive impairment | ✓ | ✓ |  | ✓ |  |  | ✓ |  |  | |
| Other and non-specific intellectual disabilities |  |  |  |  | ✓ |  |  | ✓ |  |  | |
| Memory loss |  | ✓ | ✓ |  | ✓ |  |  | ✓ |  |  | |
| *Abnormal brain activity* | |  |  |  |  |  |  | ✓ |  |  | |
| Seizures/epilepsy | Seizures and epilepsy considered in one category | ✓ | ✓ |  | ✓ |  |  | ✓ |  |  | |
| Migraines | Not considered as a separate category placed under chronic headaches | ✓ |  |  | ✓ |  |  | ✓ |  |  | |
| Chronic headaches | Includes: persistent headaches and migraines | ✓ | ✓ |  | ✓ |  |  | ✓ |  |  | |
| Vegetative state |  |  |  |  | ✓ |  |  | ✓ |  |  | |
| Other and non-specified abnormal brain activity | Includes: encephalopathy |  |  |  | ✓ |  |  | ✓ |  |  | |
| *Other and non-specified neurological disorders* | | |  |  |  |  |  | ✓ |  |  | |
| Severe neurological disorders | Includes: grouped sequelae where we cannot differentiate into the pre-defined categories | ✓ | ✓  Not precise in how they are categorised |  | ✓ |  |  | ✓ |  |  | |
| (Other and non-specified neurological disorders) Non-severe neurological disorders | Includes: neurological disabilities, severity of disability defined as mild -moderate (Halket et al. 2003 [1]) |  |  |  | ✓ |  |  | ✓ |  |  | |
| **Psychological** |  |  |  |  |  |  |  |  |  |  | |
| *Neurodevelopmental disorders* | |  |  |  | *Behavioural difficulties should be included as a subheading somewhere* | | | ✓ |  |  | |
| ADHD |  | ✓ | ✓  Can happen |  | ✓ |  |  | ✓ |  |  | |
| Autistic spectrum disorder |  | ✓ | ✓  Autism spectrum is very wide, needs to be evaluated (Autism Spectrum Rating Scales [ASRS™]) |  | ✓ |  |  | ✓ |  |  | |
| Other and non-specified neurodevelopment disorders |  |  |  |  | ✓ |  |  | ✓ |  |  | |
| *Anxiety disorders* |  |  |  |  |  |  |  | ✓ |  |  | |
| Generalised anxiety |  | ✓ | ✓ |  | ✓ |  |  | ✓ |  |  | |
| Separation anxiety |  | ✓ | ✓ |  | ✓ |  |  | ✓ |  |  | |
| Social anxiety disorder/social phobia | Includes: oppositional defiant disorder, tic disorder | ✓ | ✓ | this can also be part of a broader clinical picture | ✓ |  |  | ✓ |  |  | |
| Specific phobias |  | ✓ | Patient dependent (e.g. agoraphobia) |  | ✓ |  |  | ✓ |  |  | |
| Other and non-specific anxiety disorders |  | ? | Is sleeping disorders captured? | Consider adding sleeping disorders | ✓ |  |  | ✓ |  |  | |
| *Depressive disorders* |  |  |  |  |  |  |  |  |  |  | |
| Depression |  | ✓ | Can happen, but hard to diagnose in children (infants) and will depend on type; transient, MDD major or mild depressive disorders |  | ✓ |  |  | ✓ |  |  | |
| Other and non-specified depressive disorders |  |  |  |  | ✓ |  |  | ✓ |  |  | |
| *Trauma- and stressor-related disorders* | | |  |  |  |  |  |  |  |  | |
| Post-traumatic stress disorder |  | NA |  | Maybe for adolescents we could depict this better clinically speaking | ✓ |  |  | ✓ |  |  | |
| Other and non-specified trauma and stressor-related disorders |  |  |  |  | ✓ |  |  | ✓ |  |  | |
| *Feeding and eating disorders* | |  |  |  |  |  |  |  |  |  | |
| Eating disorder |  | ✓ | ✓  Both anorexia and obesity should be considered | Both anorexia and obesity should be considered | ✓ |  |  | ✓ |  |  | |
| Other and non-specified feeding and eating disorders |  |  |  |  | ✓ |  |  | ✓ |  |  | |
| *Disruptive, impulse-control and conduct disorders* | | |  |  |  |  |  | ✓ |  |  | |
| Oppositional defiant disorder |  | ? | Ok but difficult to say, this is usually a 'wide' neurological picture, and these symptoms are interlinked (with ADHD, MDD, autism etc) |  | ✓ |  |  | ✓ |  |  | |
| Conduct disorder |  | ? | Unclear |  | ✓ |  |  | ✓ |  |  | |
| Other and non-specified disruptive, impulse control and conduct disorders |  | NA | NA |  | ✓ |  |  | ✓ |  |  | |
| *Other and non-specific emotional/behavioural disorders* | | |  |  | ✓ |  |  | ✓ |  |  | |
| *Hydrocephalus* |  | *?* | *Not frequent* | *Not really* | ✓ |  |  | ✓ |  |  | |

**ADHD** attention deficit/hyperactivity disorder; **ARDS** Acute Respiratory Distress Syndrome; **IMD** invasive meningococcal disease; **IQ** intelligence quotient; **MDD** manic depressive disorder; **NA** not applicable

**Table S2.2. Sequelae data extraction from high-rated studies (highest SIGN rating and highest number of sequelae reported)**

| Category | Viner et al (2012), SIGN 2++  [2] | Huang et al (2020), SIGN 2+  [3] | Cabellos et al (2019), SIGN 2+  [4] | Rivero-Calle et al (2016), SIGN 2+  [5] | Borg et al (2009), SIGN 2+  [6] | Svendsen et al (2020), SIGN 2+  [7] | Gottfredson et al (2011), SIGN 2-  [8] | Sadarangani et al (2015), SIGN 2-  [9] | Stoof et al (2015), SIGN 3  [10] | Stein-Zamir et al (2014), SIGN 3  [11] | Bettinger et al (2013), SIGN 3  [12] |
| --- | --- | --- | --- | --- | --- | --- | --- | --- | --- | --- | --- |
| Number of IMD survivors (n) | 239 | 148 | 527 | 458 | 101 | 85 | 120 | 795 | 763 | 115 | 391 |
| **Physical** |  |  |  |  |  |  |  |  |  |  |  |
| Skin scarring (with/without grafting) |  | 3.66% | 3.23% | 2.62% | 17.82% |  | 1.67% | 5.79% | 3.41% |  | 6.39% |
| Other and non-specified skin disease (e.g., skin necrosis, eczema, psoriasis) |  |  |  | 1.53% |  |  | 2.40% |  |  |  |  |
| Arthritis |  |  | 7.59% |  |  |  | 5.73% |  |  |  |  |
| Limb deficiency/deformities |  |  |  |  | 3.96% |  |  | 1.38% |  | 8.70% |  |
| Amputation | 1.26%(major)  2.09% (all) | 2.44% |  | 2.18% | 2.97% |  |  | 3.65% | 3.15% | 1.74% | 3.84% |
| Other and non-specified musculoskeletal deficiencies |  |  |  | 0.44% |  |  |  | 3.14% |  |  |  |
| Renal condition |  | 7.32% | 8.92% | 1.75% |  |  | 2.77% | 2.77% | 0.79% |  | 2.05% |
| Raynaud phenomenon symptoms |  |  |  |  | 27.72% |  |  |  |  |  |  |
| Other and non-specified vascular conditions |  | 15.24% | 7.02% |  |  |  | 2.40% |  |  |  | 0.26% |
| Pulmonary condition |  |  | 10.82% | 0.44% |  |  |  |  | 0.13% |  |  |
| Other and non-specific physical conditions |  |  |  |  | 12.87% |  | 0.92% | 3.14% |  |  |  |
| **Neurological** |  |  |  |  |  |  |  |  |  |  |  |
| Blindness/severe visual impairment | 0.42% | 2.44% |  |  |  |  |  |  |  |  | 0.26% |
| Other visual impairments |  |  |  |  |  |  |  | 1.13% | 0.92% |  |  |
| Hearing loss – severe/profound bilateral (cochlear implant) | 2.45% |  |  |  | 11.88% | 2.35% |  | 5.79% |  | 2.61% | 1.53% |
| Hearing loss – moderate bilateral | 4.74% |  |  |  |  |  |  |  |  | 3.48% | 3.32% |
| Hearing loss – unilateral/hearing impairment | 6.47% | 5.49% | 4.28% | 2.62% |  | 12.94% | 2.59% |  | 5.64% | 6.96% | 2.30% |
| Palsy |  | 7.93% | 0.38% |  |  |  | 0.83% |  | 1.31% | 3.48% | 0.26% |
| Muscle weakness |  |  | 2.47% |  |  |  | 5.83% |  | 4.33% |  | 0.26% |
| Movement coordination/balance deficits |  |  |  | 0.22% | 16.83% |  | 0.00% |  | 0.13% |  |  |
| Other and non-specific motor deficits |  |  |  |  |  | 9.41% |  | 3.14% |  | 12.17% | 1.02% |
| Communication disorders | 4.18% |  |  |  | 12.87% | 4.17% |  |  | 0.39% | 12.17% |  |
| Mental retardation/low IQ | 0.84% | 2.44% |  |  |  |  |  |  | 0.52% |  |  |
| Learning disabilities |  |  |  |  |  | 11.76% |  |  |  |  |  |
| Other and non-specific intellectual disabilities |  |  |  |  |  |  | 1.48% | 0.63% |  | 22.61% |  |
| Seizures/epilepsy | 2.09% | 6.71% | 3.04% |  | 1.98% | 7.06% | 3.33% | 2.64% | 0.92% | 1.74% | 2.56% |
| Chronic headaches |  |  |  |  |  |  | 18.33% |  |  | 13.91% |  |
| Vegetative state |  |  |  |  |  |  |  |  | 0.13% |  |  |
| Other and non-specified abnormal brain activity |  |  |  |  |  |  |  |  |  |  | 0.26% |
| Severe neurological disorders |  |  |  |  |  |  |  |  | 1.18% |  | 1.02% |
| Non-severe neurological disorders |  |  |  | 5.02% |  |  |  | 1.26% |  | 12.17% |  |
| **Psychological** |  |  |  |  |  |  |  |  |  |  |  |
| ADHD | 11.41% |  |  |  |  |  |  |  |  |  |  |
| Autistic spectrum disorder | 1.34% |  |  |  |  |  |  |  |  |  |  |
| Generalised anxiety | 2.68% |  |  |  |  |  | 5.83% |  |  |  |  |
| Separation anxiety | 6.85% |  |  |  |  |  |  |  |  |  |  |
| Social anxiety disorder/social phobia | 1.34% |  |  |  |  |  |  |  |  |  |  |
| Specific phobias | 4.70% |  |  |  |  |  |  |  |  |  |  |
| Other and non-specific anxiety disorders | 0.00% |  |  |  |  |  | 6.67% |  |  |  |  |
| Depression | 0.00% |  |  |  |  |  | 5.83% |  |  |  | 0.26% |
| Post-traumatic stress disorder | 0.00% |  |  |  |  |  |  |  |  |  |  |
| Eating disorder | 0.68% |  |  |  |  |  |  |  |  |  |  |
| Oppositional defiant disorder | 11.41% |  |  |  |  |  |  |  |  |  |  |
| Conduct disorder | 6.04% |  |  |  |  |  |  |  |  |  |  |
| Other and non-specific emotional/behavioural disorders |  |  |  |  |  |  |  |  | 3.41% | 14.78% |  |
| Hydrocephalus |  | 2.44% | 0.19% |  |  | 5.88% |  |  |  |  |  |

**ADHD** attention deficit/hyperactivity disorder; **IQ** intelligence quotient; **SIGN**: Scottish Intercollegiate Guidelines Network

**Table S2.3. IMD sequelae for comprehensive map: selected percentage (range across studies) – expert validation comments**

Expert validation: **1**= Agree with sequelae extracted; **2**= Comments on proportion extracted

| Category | IMD sequelae % (range across studies) * | Source, SIGN rating | Expert validation 1 (internal RBB) ** | | Expert validation 2 (external FMT)** | | Expert validation 3 (external) | | |
| --- | --- | --- | --- | --- | --- | --- | --- | --- | --- |
| *Physical* |  |  | **1** | **2** | **1** | **2** | **1** | **2** | |
| Skin scarring (with/without grafting) | 3.66  (1.67-17.82) | Huang et al 2020, SIGN 2+ | ✓ | Under-represented, skin scaring should be at higher proportion | ✓ | I have carefully reviewed all, and I am in agreement. Not much to add. Those other concepts suggested by me in the previous section, not sure how much added value may pose to those you propose here. Only those general like quality of life, survivor dependency, family impact...which I don’t know if they are retrievable in current literature, see [13] | Important, although little data: Chronic pain, dysethesia (Gottfredson) | | ✓ |
| Other and non-specified skin disease (e.g. skin necrosis, eczema, psoriasis) | 1.53  (1.53-2.40) | Rivero-Calle 2016, SIGN 2+ | ✓ |  | ✓ |  | ✓ | | ✓ |
| Arthritis | 5.73  (5.73-7.59) | Gottfredson et al 2011, SIGN 2- | ✓ |  | ✓ |  | ✓ | | ✓ |
| Limb deficiency/deformities | 3.96  (3.96-8.70) | Borg et al 2009, SIGN 2+ | ✓ | Assume > 5% | ✓ |  | ✓ | | ✓ |
| Amputation | 1.26 (major)  2.09 (major and minor)  (1.74-3.84) | Viner et al 2012, SIGN 2++ | ✓ |  | ✓ |  | ✓ | | ✓ |
| Other and non-specified musculoskeletal deficiencies | 0.44  (0.44-3.65) | Rivero-Calle 2016, SIGN 2+ | ✓ | Under 1%? | ✓ |  | ✓ | | ✓ |
| Renal condition | 7.32  (0.79-8.92) | Huang et al 2020, SIGN 2+ | ✓ |  | ✓ |  | ✓ | | ✓ |
| Raynaud phenomenon symptoms | 27.72 | Borg et al 2009, SIGN 2+ | ✓ | Would not recommend picking such a high proportion based on only one publication. | ✓ | This one calls my attention, but I think this was specifically sought in this study and they found it, and I found it plausible | ✓ | | ✓ |
| Other and non-specified vascular conditions | 15.24  (0.26-15.24) | Huang et al 2020, SIGN 2+ | ✓ | ok, but reading the publications you have listed this is the highest proportion why are you using this one? Seems too high to me. | ✓ |  | ✓ | | ✓ |
| Pulmonary condition | 10.82  (0.13-10.82) | Cabellos et al 2019, SIGN 2+ | ✓ | same comment as above 10% vs 0.4%, (both studies are SIGN 2) | ✓ |  | ✓ | | ✓ |
| Other and non-specific physical conditions | 12.87  (0.92-12.87) | Borg et al 2009, SIGN 2+ | ✓ | same comment as above | ✓ |  | ✓ | | ✓ |
| **Neurological** |  |  |  |  |  |  |  | |  |
| Blindness/severe visual impairment | 0.42  (0.26-2.44) | Viner et al 2012, SIGN 2++ | ✓ |  | ✓ |  | ✓ | | ✓ |
| Other visual impairments | 1.13  (0.92-1.13) | Sadarangani et al 2015, SIGN 2- | ✓ |  | ✓ |  | ✓ | | ✓ |
| Hearing loss – severe/profound bilateral (cochlear implant) | 2.45  (1.53-11.88) | Viner et al 2012, SIGN 2++ | ✓ |  | ✓ |  | Cranial nerve disturbance, other than hearing in part, where facial nerve may be impactful (Gottfredson, Stoof) | | ✓ |
| Hearing loss – moderate bilateral | 4.74  (3.32-4.74) | Viner et al 2012, SIGN 2++ | ✓ |  | ✓ |  |  | | ✓ |
| Hearing loss – unilateral/hearing impairment | 6.47  (2.30-12.94) | Viner et al 2012, SIGN 2++ | ✓ | OK, but here you did not pick 12% (of Svendson SIGN 2+), can one SIGN 2++ be more important than several SIGN 2+ ? | ✓ |  | ✓ | | ✓ |
| Palsy | 0.26  (0.26-7.93) | Bettinger 2013*, SIGN 3 | ✓ |  | ✓ |  | ✓ | | ✓ |
| Muscle weakness | 2.47  (0.26-5.83) | Cabellos et al 2019, SIGN 2+ | ✓ |  | ✓ |  | ✓ | | ✓ |
| Movement coordination/balance deficits | 0.22  (0.00-16.83) | Rivero-Calle 2016, SIGN 2+ | ✓ |  | ✓ |  | ✓ | | ✓ |
| Other and non-specific motor deficits | 1.02  (1.02-12.17 | Bettinger 2013*, SIGN 3 | ✓ |  | ✓ |  | ✓ | | ✓ |
| Communication disorders | 4.18  (0.39-12.17) | Viner et al 2012, SIGN 2++ | ✓ | Same comment as above, you did not pick 12% | ✓ |  | ✓ | | ✓ |
| Mental retardation/low IQ | 0.84  (0.52-2.44) | Viner at al 2012, SIGN 2++ | ✓ | Would rename this to under-development, so something similar vs retardation | ✓ |  | ✓ | | ✓ |
| Learning disabilities | 11.76 | Svendsen et al 2020, SIGN 2+ | ✓ | Interesting to have just one reference reporting this… (this is important) | ✓ |  | ✓ | | ✓ |
| Other and non-specific intellectual disabilities | 1.48  (0.63-22.61) | Gottfredson et al 2011, SIGN 2- | ✓ | Vague, but ok | ✓ |  | ✓ | | ✓ |
| Seizures/epilepsy | 2.09  (0.92-7.06) | Viner et al 2012, SIGN 2++ | ✓ |  | ✓ |  | ✓ | | ✓ |
| Chronic headaches | 13.91  (13.91-18.33) | Stein-Zamir et al 2014, SIGN 3 | ✓ | ok, (in section 2 you have also listed migraine, and here just chronic headaches, why ?) | ✓ |  | ✓ | | ✓ |
| Vegetative state | 0.13 | Stoof et al 2015, SIGN 3 | 🗶 | No - seems too extreme | ✓ |  | ✓ | | ✓ |
| Other and non-specified abnormal brain activity | 0.26 | Bettinger 2013*, SIGN 3 | ✓ | ok, but not precise, difficult to evaluate the relevance | ✓ |  | ✓ | | ✓ |
| Severe neurological disorders | 1.02  (1.02-1.18) | Bettinger 2013*, SIGN 3 | ✓ |  | ✓ |  | ✓ | | ✓ |
| Non-severe neurological disorders | 5.02  (1.26-12.17) | Rivero-Calle 2016, SIGN 2+ | ✓ | ok (same comment 5% vs 12%) | ✓ |  | ✓ | | ✓ |
| **Psychological** |  |  |  |  |  |  |  | |  |
| ADHD | 11.41 | Viner et al 2012, SIGN 2++ | ✓ |  | ✓ |  | ✓ | | ✓ |
| Autistic spectrum disorder | 1.34 | Viner et al 2012, SIGN 2++ | ✓ |  | ✓ |  | ✓ | | ✓ |
| Generalised anxiety | 2.68  (2.68-5.83) | Viner et al 2012, SIGN 2++ | ✓ |  | ✓ |  | ✓ | | ✓ |
| Separation anxiety | 6.85 | Viner et al 2012, SIGN 2++ | ✓ |  | ✓ |  | ✓ | | ✓ |
| Social anxiety disorder/social phobia | 1.34 | Viner et al 2012, SIGN 2++ | ✓ |  | ✓ |  | ✓ | | ✓ |
| Specific phobias | 4.70 | Viner et al 2012, SIGN 2++ | ✓ |  | ✓ |  | ✓ | | ✓ |
| Other and non-specific anxiety disorders | 6.67  (0.00-6.67) | Gottfredson et al 2011, SIGN 2- | ? | Not specific enough. how you pick 6%, when you have 0% for [4] with high SIGN? | ✓ |  | ✓ | | ✓ |
| Depression | 0.26  (0.00-5.83) | Bettinger 2013*, SIGN 3 | ✓ |  | ✓ |  | ✓ | | ✓ |
| Post-traumatic stress disorder | 0.00 | Viner et al 2012, SIGN 2++ | 🗶 | NA | ✓ |  | ✓ | | ✓ |
| Eating disorder | 0.68 | Viner et al 2012, SIGN 2++ | ✓ |  | ✓ |  | ✓ | | ✓ |
| Oppositional defiant disorder | 11.41 | Viner et al 2012, SIGN 2++ | ✓ | limitations - 11% is high based on only one manuscript | ✓ |  | ✓ | | ✓ |
| Conduct disorder | 6.04 | Viner et al 2012, SIGN 2++ | ? | Unclear | ✓ |  | ✓ | | ✓ |
| Other and non-specific emotional/behavioural disorders | 3.41 | Stoof et al 2015, SIGN 3 | NA |  | ✓ |  | ✓ | | ✓ |
| Hydrocephalus | 2.44 | Huang et al 2020, SIGN 2+ | ✓ | 2.44% is high | ✓ |  | ✓ | | ✓ |

**ADHD** attention deficit/hyperactivity disorder; **IMD** invasive meningococcal disease; **IQ** intelligence quotient; **NA** not applicable; **SIGN**: Scottish Intercollegiate Guidelines Network

* Point estimates from studies with highest SIGN rating and highest number of outcomes reported, range from across studies reporting sequela; **Cells highlighted in grey indicate that experts expressed uncertainty about inclusion in the sequelae map. Cells highlighted in yellow indicate that expert 1 agreed with inclusion but questioned the selected % as several studies reported different % sequelae and had good SIGN ratings.

General comment (Expert 3): Data selection seems representative/reliable, yet ‘healthy’ controls are usually missing, which makes interpretation of rather subtle neurological sequelae difficult (with respect to quantity). Also, studies should be separately analysed for children and adults where possible.

**Table S2.4. IMD sequelae relevant for economic evaluation: selected percentage (range across studies) – expert validation comments**

Expert validation: **1**= Agree with sequelae extracted; **2**= Comments on proportion extracted

| Category | IMD sequelae % (range across studies) * | Source, SIGN rating | Expert validation 1 (internal) | | Expert validation 2 (external)** | | Expert validation 3 (external)** | |
| --- | --- | --- | --- | --- | --- | --- | --- | --- |
| **Physical** |  |  | **1** | **2** | **1** | **2** | **1** | **2** |
| Skin scarring (with/without grafting) | 3.66  (1.67-17.82) | Huang et al 2020, SIGN 2+ | ✓ |  | ✓ |  | ✓ |  |
| Other and non-specified skin disease (e.g. skin necrosis, eczema, psoriasis) | 1.53  (1.53-2.40) | Rivero-Calle 2016, SIGN 2+ | ✓ |  | ✓ |  | ✓ |  |
| Amputation | 1.26 (major)  2.09 (major and minor)  (1.74-3.84) | Viner et al 2012, SIGN 2++ | ✓ |  | ✓ |  | ✓ |  |
| Renal condition | 7.32  (0.79-8.92) | Huang et al 2020, SIGN 2+ | ✓ | ok, I do not see any more pulmonary conditions, (could be more precise), if the patient becomes more susceptible to respiratory infections, like flu, RSV… this has an economic impact | ✓ |  | ✓ |  |
| **Neurological** |  |  |  |  |  |  |  |  |
| Blindness/severe visual impairment | 0.42  (0.26-2.44) | Viner et al 2012, SIGN 2++ | ✓ |  | ✓ |  | ✓ |  |
| Hearing loss – severe/profound bilateral (cochlear implant) | 2.45  (1.53-11.88) | Viner et al 2012, SIGN 2++ | ✓ |  | ✓ |  | ✓ |  |
| Hearing loss – moderate bilateral | 4.74  (3.32-4.74) | Viner et al 2012, SIGN 2++ | ✓ |  | ✓ |  | ✓ |  |
| Hearing loss – unilateral/hearing impairment | 6.47  (2.30-12.94) | Viner et al 2012, SIGN 2++ | ✓ |  | ✓ |  | ✓ |  |
| Motor deficits (composite of those identified) | 3.97 (composite of palsy, muscle weakness, movement coordination and other non-specified)  (1.54-34.83) | Bettinger 2013*, SIGN 3  Rivero-Calle 2016, SIGN 2+  Cabellos et al 2019, SIGN 2+ | ✓ | Ok to assume motor deficits as the composite of palsy, muscle weakness, movement co-ordination and other non-specified motor deficits | ✓ | Yes, I think so | ✓ |  |
| Communication disorders | 4.18  (0.39-12.17) | Viner et al 2012, SIGN 2++ | ✓ |  | ✓ |  | ✓ |  |
| Mental retardation/low IQ | 0.84  (0.52-2.44) | Viner at al 2012, SIGN 2++ | ✓ |  | ✓ |  | ✓ |  |
| Seizures/epilepsy | 2.09  (0.92-7.06) | Viner et al 2012, SIGN 2++ | ✓ |  | ✓ |  | ✓ |  |
| Severe neurological disorders | 1.02  (1.02-1.18) | Bettinger 2013*, SIGN 3 | ✓ |  | ✓ |  | ✓ |  |
| Non-severe neurological disorders | 5.02  (1.26-12.17) | Rivero-Calle 2016, SIGN 2+ | ✓ | For an economic evaluation this will need to be more precise, to understand the potential impact | ✓ |  | ✓ |  |
| **Psychological/behavioural** |  |  |  |  |  |  |  |  |
| ADHD | 11.41  (-) | Viner et al 2012, SIGN 2++ | ✓ |  | ✓ |  | ✓ |  |
| Generalised anxiety | 2.68  (2.68-5.83) | Viner et al 2012, SIGN 2++ | ✓ |  | ✓ |  | ✓ |  |
| Separation anxiety | 6.85  (-) | Viner et al 2012, SIGN 2++ | ✓ |  | ✓ |  | ✓ |  |
| Depression | 0.26  (0.00-5.83) | Bettinger 2013*, SIGN 3 | ✓ | Depression is not very precise, MDD may have a societal and economic impact, but unsure for 'depression', and how about learning disabilities? | ✓ |  | ✓ |  |

**ADHD** attention deficit/hyperactivity disorder; **IMD** invasive meningococcal disease; **IQ** intelligence quotient; **MDD** manic depressive disorder; **RSV** Respiratory syncytial virus; **SIGN**: Scottish Intercollegiate Guidelines Network

* Point estimates from studies with highest SIGN rating and highest number of outcomes reported, range from across studies reporting sequela

**References**

1. Halket S, de Louvois J, Holt DE, Harvey D. Long term follow up after meningitis in infancy: behaviour of teenagers. Arch Dis Child. 2003;88(5):395-8. <https://doi.org/10.1136/adc.88.5.395>

2. Viner RM, Booy R, Johnson H, Edmunds WJ, Hudson L, Bedford H et al. Outcomes of invasive meningococcal serogroup B disease in children and adolescents (MOSAIC): a case-control study. Lancet Neurol. 2012;11(9):774-83. <https://doi.org/10.1016/S1474-4422(12)70180-1>

3. Huang L, Heuer OD, Janßen S, Häckl D, Schmedt N. Clinical and economic burden of invasive meningococcal disease: Evidence from a large German claims database. PLoS ONE. 2020;15(1):e0228020. <https://doi.org/10.1371/journal.pone.0228020>

4. Cabellos C, Pelegrín I, Benavent E, Gudiol F, Tubau F, Garcia-Somoza D et al. Invasive meningococcal disease: What we should know, before it comes back. Open Forum Infect Dis. 2019;6(3):ofz059. <https://doi.org/10.1093/ofid/ofz059>

5. Rivero-Calle I, Vilanova-Trillo L, Pardo-Seco J, Salvado LB, Quinteiro LI, Martinon-Torres F et al. The burden of pediatric invasive meningococcal disease in Spain (2008-2013). Pediatr Infect Dis J. 2016;35(4):407-13. <https://doi.org/10.1097/INF.0000000000001048>

6. Borg J, Christie D, Coen PG, Booy R, Viner RM. Outcomes of meningococcal disease in adolescence: prospective, matched-cohort study. Pediatrics. 2009;123(3):e502-e9. <https://doi.org/10.1542/peds.2008-0581>

7. Svendsen MB, Ring Kofoed I, Nielsen H, Schønheyder HC, Bodilsen J. Neurological sequelae remain frequent after bacterial meningitis in children. Int J Pediatr. 2020;109(2):361-7. <https://doi.org/10.1111/apa.14942>

8. Gottfredsson M, Reynisson IK, Ingvarsson RF, Kristjansdottir H, Nardini MV, Sigurdsson JF et al. Comparative long-term adverse effects elicited by invasive group B and C meningococcal infections. Clin Infect Dis. 2011;53(9):e117-e24. <https://doi.org/10.1093/cid/cir500>

9. Sadarangani M, Scheifele DW, Halperin SA, Vaudry W, Le SN, Tsang R et al. Outcomes of invasive meningococcal disease in adults and children in Canada between 2002 and 2011: a prospective cohort study. Clin Infect Dis. 2015;60(8):e27-e35. <https://doi.org/10.1093/cid/civ028>

10. Stoof SP, Rodenburg GD, Knol MJ, Rümke LW, Bovenkerk S, Berbers GA et al. Disease Burden of Invasive Meningococcal Disease in the Netherlands Between June 1999 and June 2011: A Subjective Role for Serogroup and Clonal Complex. Clin Infect Dis. 2015;61(8):1281-92. <https://doi.org/10.1093/cid/civ506>

11. Stein-Zamir C, Shoob H, Sokolov I, Kunbar A, Abramson N, Zimmerman D. The clinical features and long-term sequelae of invasive meningococcal disease in children. Pediatr Infect Dis J. 2014;33(7):777-9. <https://doi.org/10.1097/INF.0000000000000282>

12. Bettinger JA, Scheifele DW, Le Saux N, Halperin SA, Vaudry W, Tsang R et al. The disease burden of invasive meningococcal serogroup B disease in Canada. Pediatr Infect Dis J. 2013;32(1):e20-e5. <https://doi.org/10.1097/INF.0b013e3182706b89>

13. Olbrich KJ, Müller D, Schumacher S, Beck E, Meszaros K, Koerber F. Systematic Review of Invasive Meningococcal Disease: Sequelae and Quality of Life Impact on Patients and Their Caregivers. Infect Dis Ther. 2018;7(4):421-38. <https://doi.org/10.1007/s40121-018-0213-2>
